# Effect of Lumican on the Migration of Human Mesenchymal Stem Cells and Endothelial Progenitor Cells: Involvement of Matrix Metalloproteinase-14

**DOI:** 10.1371/journal.pone.0050709

**Published:** 2012-12-07

**Authors:** Mariusz Malinowski, Katarzyna Pietraszek, Corinne Perreau, Mateusz Boguslawski, Véronique Decot, Jean-François Stoltz, Laurent Vallar, Jolanta Niewiarowska, Czeslaw Cierniewski, François-Xavier Maquart, Yanusz Wegrowski, Stéphane Brézillon

**Affiliations:** 1 Laboratoire de Biochimie Médicale et de Biologie Moléculaire, CNRS FRE 3481, Université de Reims Champagne-Ardenne, Reims, France; 2 Institute of Medical Biology, Polish Academy of Sciences, Lodz, Poland; 3 Unité de Thérapie Cellulaire et banque de Tissus, CHU Brabois, CNRS UMR 7561, Faculté de Médecine, Vandoeuvre Lès Nancy, France; 4 Microarray Center, CRP-Santé, Luxembourg, Luxembourg; 5 Department of Molecular and Medical Biophysics, Medical University of Lodz, Lodz, Poland; 6 CHU de Reims, Reims, France; University of Patras, Greece

## Abstract

**Background:**

Increasing number of evidence shows that soluble factors and extracellular matrix (ECM) components provide an optimal microenvironment controlling human bone marrow mesenchymal stem cell (MSC) functions. Successful *in vivo* administration of stem cells lies in their ability to migrate through ECM barriers and to differentiate along tissue-specific lineages, including endothelium. Lumican, a protein of the small leucine-rich proteoglycan (SLRP) family, was shown to impede cell migration and angiogenesis. The aim of the present study was to analyze the role of lumican in the control of MSC migration and transition to functional endothelial progenitor cell (EPC).

**Methodology/Principal Findings:**

Lumican inhibited tube-like structures formation on Matrigel® by MSC, but not EPC. Since matrix metalloproteinases (MMPs), in particular MMP-14, play an important role in remodelling of ECM and enhancing cell migration, their expression and activity were investigated in the cells grown on different ECM substrata. Lumican down-regulated the MMP-14 expression and activity in MSC, but not in EPC. Lumican inhibited MSC, but not EPC migration and invasion. The inhibition of MSC migration and invasion by lumican was reversed by MMP-14 overexpression.

**Conclusion/Significance:**

Altogether, our results suggest that lumican inhibits MSC tube-like structure formation and migration *via* mechanisms that involve a decrease of MMP-14 expression and activity.

## Introduction

Bone marrow mesenchymal stem cells (MSC) are multipotent non-hematopoietic progenitor cells that support hematopoiesis through the secretion of growth factors and cytokines. They are heterogeneous as defined by their adherence to plastic, self-renewal capabilities, and ability to differentiate into osteoblasts, adipocytes, or chondrocytes. They are good candidates for cell therapy because of their differentiating potential, limited tendency to produce tumors, ease of isolation, immunologically privileged nature, and ability to home to damaged tissues [1]. MSC respond to a damage signal by escaping from their niche into circulation, then adhering to and transmigrating across endothelium, invading extracellular matrix (ECM), and finally engrafting into target tissue, where they proliferate and differentiate. Besides the capacity to differentiate, the ability to migrate is an imperative requirement for stem cells to accomplish their biological functions. The molecular mechanisms for MSC recruitment and migration are not fully defined. The fairly well characterized process is the release of hematopoietic stem cells from their niche in the bone marrow into circulation. To facilitate such mobilization, in response to specific signal transduction cascades, various metalloproteases (MMPs) are secreted, such as MMP-2/−9, which degrade the basement membrane. Recently, regulatory role of MMP-14, membrane-type 1 MMP (MT1-MMP), in MSC trafficking through the interstitial ECM was explored [2]. It can be assumed that similar MMP-dependent mechanisms are utilized in the mobilization of endothelial progenitor cell (EPC) [3], [4].

MSC are thought to occupy a perivascular niche, which is a prime location for regulating vessel stability. MSC can be differentiated *in vitro* into EPC by a protocol based on low-serum culture supplemented with vascular endothelial growth factor (VEGF) [5], [6]. Under these conditions, MSC acquire several features of mature endothelium [5]. Thus, bone marrow-derived EPC could either be involved in malignant processes such as angiogenic switch in tumor growth, tumor neovascularization or metastatic progression [7] or have a beneficial effect during myocardial ischemia, infarction and wound healing. In response to proangiogenic factors, EPC could mobilize, move to the target destination and incorporate directly into neovessels [8]. MSC may also contribute to a vascular niche by providing growth factors rather than by incorporating into vascular structures. Bone marrow MSC can provide a local environment that favors migration and vascularisation of the surroundings of injured sites [9]. Therefore, MSC may be considered as an alternative source of endothelial progenitors for clinical therapies like tissue replacement or vascularization of artificial organs.

Recently, it has become widely considered that ECM present in the stem cell niche provides optimal microenvironment to sustain and control their physiological functions, like mobilization, migration, homing or differentiation [10]. Two members of small leucine rich proteoglycan (SLRP) family, biglycan and decorin, were shown to be essential for maintaining an appropriate number of mature osteoblasts by modulating the proliferation and survival of bone marrow MSC [11].

Lumican, a 37 kDa core protein proteoglycan belonging to the SLRP family [12], is expressed in different tissues [13]–[15]. In addition to its role in the regulation of type I collagen fibrillogenesis [16], lumican was shown to control cancer progression [17]. Previous data from our laboratory demonstrated direct anti-tumor properties of lumican in melanoma [18], [19]. Lumican was able to inhibit melanoma cell migration *via* alteration of the actin network and focal adhesion complexes [20]. The inhibition of the cell migration was mediated by α2β1 integrin, to which lumican binds directly [21], [22].

In addition, we showed that lumican exhibited angiostatic properties and inhibited endothelial cell invasion, angiogenic sprouting, and vessel formation in mice [23]. Endothelial cell migration and blood vessels density in lung metastatic nodules were shown to be significantly inhibited in presence of lumican [24]. The aim of the present study was to analyze the role of lumican in the control of MSC migration and transition to functional EPC. Our results indicate that lumican did not impair MSC differentiation to EPC. Lumican inhibited MSC tube-like formation and migration in MSC by a concomitant decrease of MMP-14 expression and activity, but had no effect on EPC.

## Materials and Methods

### Ethics Statement

Collection and utilization of human mesenchymal stem cells and tissues for research were approved to the Unité de Thérapie Cellulaire et Tissus (UTCT) by the French Ministry of Scientific Research (Authorization AC 2008-449). A written informed consent was obtained from all participants involved in the study.

Collection and utilization of human skin biopsies were approved by the Institutional Review Board of the Reims University Hospital (CHU de Reims) and a written informed consent was obtained from all patients.

### Cell Culture

MSC were prepared in the Unité de Thérapie Cellulaire et banque de Tissus (CHU Brabois, Vandoeuvre Lès Nancy, France). Bone marrow samples, aspirated from the iliac crest, were obtained from healthy donors. Bone marrow cells were initially plated in αMEM medium (Lonza, Verviers, Belgium) at the concentration of 7.5×10^4^/cm^2^. After 4–48 hours, growth medium and non-adherent cells were discarded. MSC were expanded in αMEM medium supplemented with 1.2 µg/µl basic Fibroblast Growth Factor (bFGF). MSC were cultured up to five passages. MSC to EPC differentiation and cell culture on different coatings of ECM proteins are described in Material S1. Human fibrosarcoma cell line (HT-1080, CCL-121™, ATCC), human cervical cancer cells (HeLa, CCL-2™, ATCC), human dermal fibroblasts and Human Umbilical Vascular Endothelial Cells (HUVEC, C-12200, Promocell) were cultured as recommended by the suppliers.

### Tube-like Formation Assays

Matrigel® (BD Biosciences) (10 mg/mL) was coated on a 24-well culture plate, (Nunc, Roskilde, Denmark), (300 µL per well). After 30 min of Matrigel® setting at 37°C, cells (5×10^4^) were seeded onto the gel in a serum-free medium. When needed, recombinant human tissue inhibitors of metalloproteinases (TIMPs) -1, -2 and -3 (200 ng/mL) or lumican (57 kDa) (100 nM) were added to the cell culture medium. Tube-like structures were observed after 24 h of cell incubation at 37°C with a phase-contrast microscope (Axiovert 25, Zeiss) and images were taken. The semi-quantitative evaluation of the tube-like length in ten randomly selected fields was performed using ImageJ software and NeuronJ plug-in tool [24]. Experiments were performed in triplicate on three different donors.

### MMP-14 Activity Assays

MSC or EPC were seeded for 7 or 21 days, respectively, on uncoated culture plates or ECM coatings. MMP-14 activity was measured using a SensoLyte® 520 MMP-14 Assay Kit (AnaSpec, San Jose, USA). The activity of MMP-14 in MSC and EPC samples was measured according to the protocol of the supplier.

### *In vitro* Migration Assays

The migration assay was performed using culture-inserts (Biovalley, Marne-la-Vallée, France) composed of 2 chambers separated by a “wall”. After withdrawing of the insert, the empty space left by the “wall” simulates a wound and enables the cells to migrate. Cells were seeded on 12-well plates in culture-inserts with 15×10^3^ cells per chamber in 70 µL of complete cell culture medium. Twenty four hours after incubation at 37°C, the culture inserts were removed, cells were rinsed twice with PBS and the wells were filled with 2 mL of serum-free cell culture medium. When needed, lumican (57 kDa) was added at a final concentration of 100 nM. Cell motility was followed using an inverted microscope (Axiovert 200M; Zeiss, Oberkoken, Germany) equipped with a transparent environmental chamber (Climabox; Zeiss) with 5% (v/v) CO_2_ in air at 37°C. The microscope was driven by the Metamorph® software (Roper Scientific, Evry, France). The cell position was recorded with a charge-coupled device camera (CoolsnapHQ: Roger Scientific) during 48 hours at 30 min intervals. Cells from 3 independent experiments (10 randomly selected single cells per microscopic field, 3 microscopic fields per insert, 3 replicate inserts for each condition) were mapped, their average migration speed was quantified using the Qmig-2D software [24]. See Materials S1 for more details.

### *In vitro* Invasion Assays

ThinCert™ cell culture inserts (24-well, pore size 8 µm; Greiner Bio-One, Courtaboeuf, France) were seeded with 50,000 cells in 200 µL of MSC or EPC medium containing 0.5% BSA. When needed, 100 nM lumican (57 kDa) was applied to the upper chamber at the time of seeding. Inserts were pre-coated with 50 µg Matrigel® (BD Biosciences) gelled at 37°C for 1 h. Eight hundred µL of medium with 10% or 2% FBS were added to the lower chamber and served as a chemotactic agent for MSC or EPC, respectively. Negative control medium contained 2% BSA. After 48 h of incubation, non-invading cells were wiped off from the upper side of the membrane and cells on the lower side were fixed in 4% paraformaldehyde (20 min at room temperature). Invasion of MSC and EPC was determined by counting the number of Hoechst 33342 (5 µg/mL, Invitrogen)-stained nuclei on the lower side of the membrane under ×200 magnification using a inverted microscope (Zeiss Axiovert-25) equipped with a digital camera. Each individual experiment (n = 3) had triplicate inserts and three microscopic fields were counted per insert. See Material S1 for more details.

### Statistical Analyses

Results were expressed as mean ± S.D. Statistical significance between groups was assessed by unpaired Student’s *t*-test. Differences with *p*<0.05 were considered significant.

## Results

### Characterization of MSC and EPC

**Figure 1:**
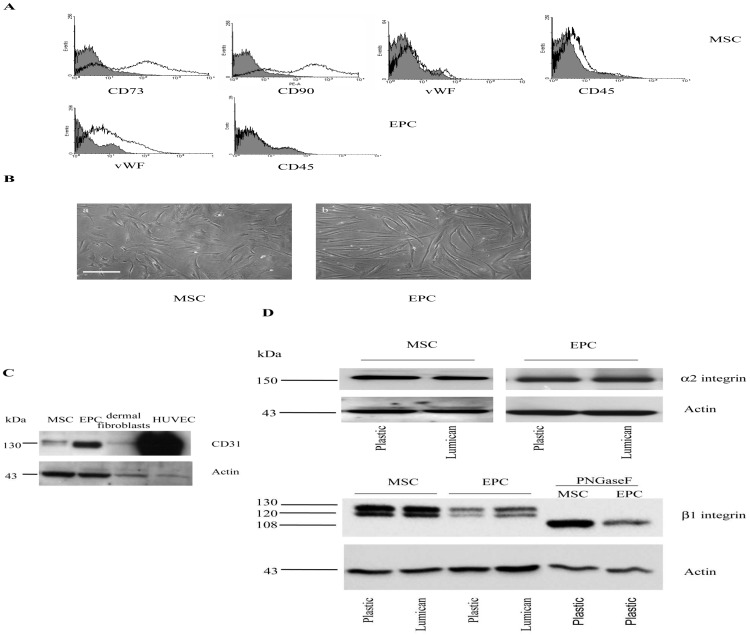
Characterization of MSC and EPC. (A)**:** Flow cytometry analysis. MSC and EPC were analyzed for expression of endothelial cell-specific marker vWF and mesenchymal stem cell markers CD73 and CD90. CD45, a typical hematopoietic marker, was used as negative control. Filled areas show isotype control staining, empty areas represent specific marker expression. (B): MSC morphology was heterogeneous with spread, star-shaped cells but also fibroblast-like cells (a). *In vitro* differentiated EPC exhibited a more elongated shape (b). Scale bars: 150 µm (a, b). (C): CD31 expression in MSC, EPC, dermal fibroblasts and HUVEC**.** Cell lysates were analyzed for the expression of endothelial cell-specific marker CD31 by Western blotting with specific monoclonal antibody. β-actin antibody was used to compare protein loading. The migration position of the bands and the name of the protein are given on the left and right margins, respectively. (D): Cell lysates (30 µg) of MSC and EPC from uncoated or lumican-coated (30 µg/cm^2^) cultures were resolved by SDS polyacrylamide gel electrophoresis. Protein levels of α2 and β1 integrins were determined by Western blotting. N-glycosidase F (PNGaseF) digestion was performed on MSC and EPC samples to deglycosylate the glycosylated forms of β1 integrins. Loading and transfer were shown by probing with anti-β-actin antibody.

To differentiate MSC into EPC, MSC were incubated in endothelial basal medium containing 50 ng/ml of VEGF for 13 days. Cells were tested for the presence of specific markers for MSC (CD73, CD90) and endothelial cells (CD31, von Willebrand factor (vWF)). As evaluated by flow cytometry (Figure 1A), MSC expressed very high levels of CD73 and CD90 (2 log shifts compared to the negative isotype controls) and did not express vWF and CD45 hematopoietic marker. EPC expressed vWF and were negative for CD45.

There was a significant difference in MSC and EPC morphology (Figure 1B). Namely MSC formed a heterogeneous cell population with spread, star-shaped cells but also fibroblast-like cells at low cell density (Figure 1Ba), whereas EPC appeared as elongated, spindle-shaped cells (Figure 1Bb).

The expression of CD31 in MSC, EPC, dermal fibroblasts and HUVEC was analyzed by Western blotting using anti-CD31 antibody (Figure 1C). Expression of CD31 was increased in EPC, when compared to that of MSC and was similar in control EPC (differentiated on plastic) and in EPC differentiated on lumican (not shown). Lumican did not influence the expression of CD31, indicating that the VEGF-induced differentiation of MSC to EPC phenotype was not inhibited by the presence of this protein.

In order to better characterize and to distinguish between MSC and EPC, we turned to genome-wide expression profiles by using Affymetrix GeneChip^®^. A total of 63 different genes were found differentially expressed in EPC as compared to MSC. The twelve most down- and up-regulated gene transcripts (False Discovery Rate (FDR) = 0.05) in EPC *versus* MSC are shown in supplemental material (Table S1). These data validated that MSC and EPC were two distinct cell populations.

Alpha2 integrin, to which lumican binds directly, was detected at a similar level of expression in MSC and EPC by Western immunoblotting (Figure 1D). The expression of α2 integrin subunit was not modified when cells were cultured on lumican-coated dishes, as compared to plastic (Figure 1D).

The polyclonal anti-human β1 integrin detected two bands in the MSC and EPC cell extracts, with an approximate size of 130 kDa and 120 kDa in reducing conditions. These two bands correspond to different glycosylated forms of β1 integrin, as confirmed by the digestion with N-glycosidase F (PNGaseF), which cleaves only at the N-linkage, of the MSC and EPC cell extracts. After digestion, the 130 kDa and 120 kDa bands were no longer detected but instead a 108 kDa band was revealed in MSC and EPC cell extracts (Figure 1D). MSC exhibited a higher level of β1 integrin expression than EPC. No significant difference in β1 integrin expression (130 kDa and 120 kDa bands) could be observed in presence or in absence of lumican in MSC. In EPC, the β1 integrin expression was slightly increased in presence of lumican The distribution of actin cytoskeleton, which is altered in melanoma cells in presence of lumican [25], was not significantly remodelled in MSC and EPC in comparison to other ECM substrata (supplemental material, Figure S1). Moreover, we investigated by Western immunoblotting (supplemental material, Figure S2) the ratio of FAK-pY397 to total FAK in MSC and EPC incubated for 15 min with or without 100 nM lumican. The ratio of FAK-pY397 to total FAK was not significantly different in presence or in absence of lumican, both in MSC and EPC (Figure S2).

### Lumican does not Alter Proliferation Rate and Apoptosis in MSC and EPC

Since previous data reported that lumican induced apoptosis on human endothelial cells [26], we checked if lumican exerted any cytotoxic effects on MSC and EPC. For that purpose, trypan blue exclusion assay was performed to check the viability of the cells. It yielded values above 92% of living cells in all experiments, demonstrating no cytotoxic effects. We also observed no effect on cell proliferation, as estimated by 3-[4,5-dimethylthiazol-2-yl]-2,5-diphenyltetrazolium bromide (MTT) method (supplemental material, Figure S3A), and no apoptosis induction, as evaluated by Hoechst staining (supplemental material, Figure S3B) after 7 days (MSC) or 21 days (EPC) of culture on uncoated or lumican-coated plates. These results were confirmed by Western blotting with antibodies raised against cyclin D1, Bax, Bcl-2 and Fas receptor (supplemental material, Figure S3C). In our case, the latter was more expressed in MSC than in EPC. Moreover, the Bax/Bcl-2 ratio evaluated in both cell types did not change between ECM substrata (supplemental material, Figure S3D). These results indicated that lumican did not induce cell death of MSC and EPC.

### Lumican Impairs Tube-like Structure Formation by MSC

**Figure 2:**
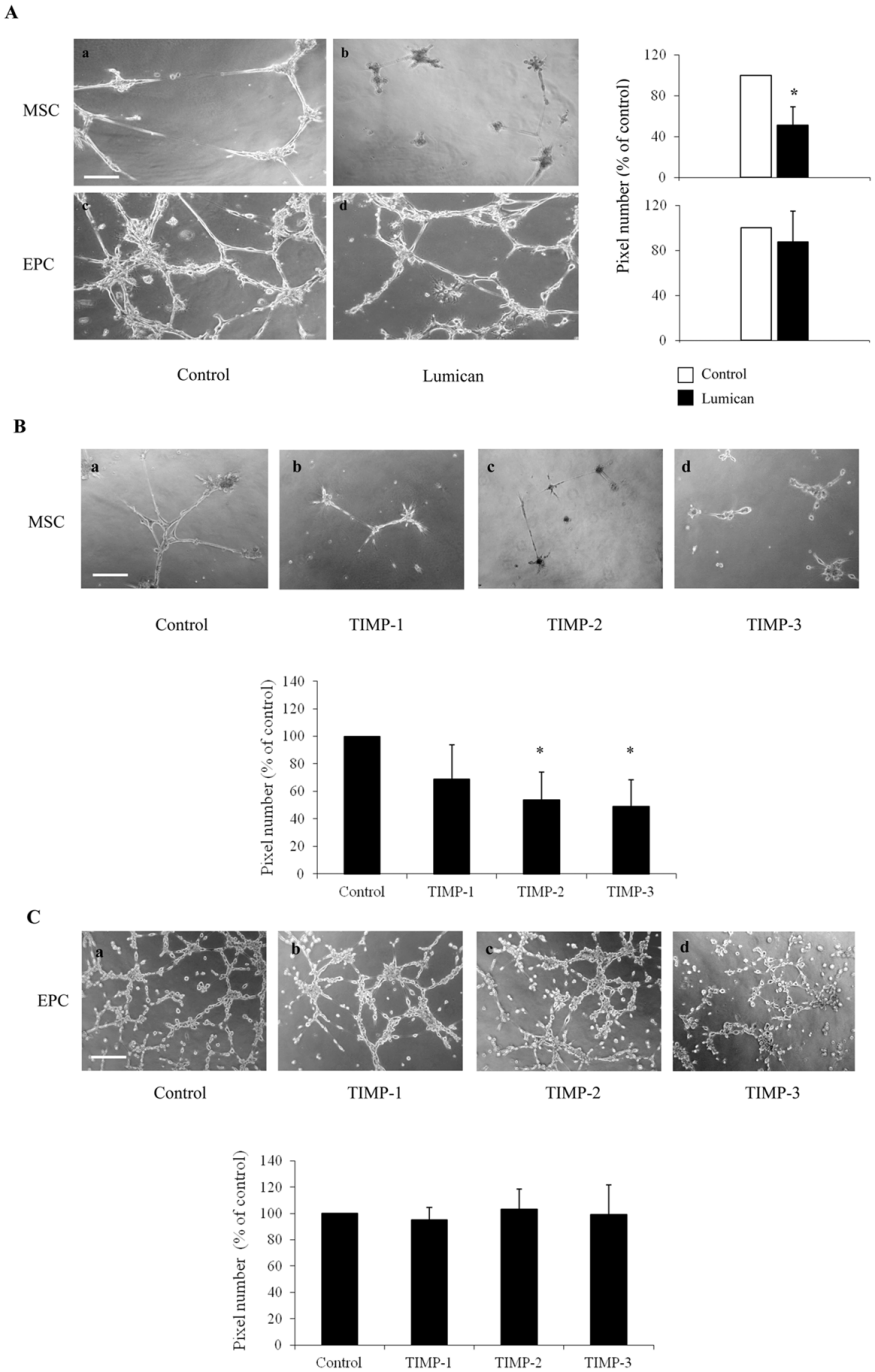
Lumican inhibits MSC tube-like formation. (A): Lumican inhibits *in vitro* tube-like formation by MSC but not EPC. Tube formation on Matrigel® (a-d) in control (a, c) or 100 nM lumican-supplemented medium (b, d) was observed twenty four hours after MSC (a, b) and EPC (c, d) seeding. Representative photographs are presented on the left panel. The semi-quantitative evaluation of the tube network from ten randomly selected fields was performed using ImageJ software and NeuronJ plugin (upper right diagram for MSC, lower right diagram for EPC). Experiments were performed in triplicate on three different donors. Results represent the mean ± S.D. Scale bar: 180 µm *p<0.05. (B, C): MSC and EPC tube-like structure formation in presence of TIMPs. Tube formation of MSC (B) or EPC (C) on Matrigel®, in control condition (a), with 200 ng/ml of TIMP-1 (b), TIMP-2 (c), TIMP-3 (d) in cell culture medium 24 h after seeding. Representative photographs are presented on the upper panels of the figure. The semi-quantitative evaluation of the branch network (lower diagrams) was performed as described above. Results represent the mean ± S.D. Scale bar: 180 µm *p<0.05.

Bone marrow-derived MSC were described to be a source of EPC, contributing to tumor angiogenesis [27]. As lumican was shown to be involved in the control of angiogenesis and particularly tumor angiogenesis [23], [24], the ability of MSC and EPC to form tube-like structures was investigated *in vitro* (Figure 2A). When grown on Matrigel®, both MSC and EPC spontaneously acquired an elongated morphology and formed a capillary network within the gel after 24 hours (Figure 2Aa, 2Ac). The tube-like structure network formed by MSC was significantly decreased (−50%) in presence of 100 nM lumican (Figure 2Ab). In contrast to MSC, EPC exhibited a more extended network (Figure 2Ac) which was not significantly impaired by the presence of 100 nM lumican (Figure 2Ad).

Gallardin®, a MMP inhibitor, blocked the formation of the tube-like strutures by MSC but not EPC (supplemental material, Figure S4), while aprotinin, a serine proteinase inhibitor, had no effect on this process, regardless of the cell type used (not shown). This suggested that MMPs, but not serine proteinases, were involved in the regulation of tube-like structure formation by MSC, but not EPC, confirming a cell type differential effect of lumican.

Dermal fibroblasts are known to form tubular structures when cultured on Matrigel®. A 24 h incubation with 100 nM lumican did not impair the tube-like formation (supplemental material, Figure S5). Thus, lumican inhibition of tube-like formation of MSC, but not dermal fibroblasts and EPC, does not simply rely on a mesenchymal feature *versu*s an endothelial feature.

### TIMP-2 and TIMP-3 Inhibit Tube-like Structure Formation in MSC

MMP activity is controlled by different TIMPs [28], and its tight balance has been shown to be critical during capillary morphogenesis [29]. As such, we tested the ability of TIMPs to inhibit tube-like structure formation in MSC. The addition of TIMP-1 (200 ng/ml), which has a relatively low affinity for the MT-MMPs [28], did not significantly suppress the tube-like structure formation (Figure 2Bb). In contrast, TIMP-2 or TIMP-3, described to inhibit secreted MMPs as well as MT-MMPs [28], were able to efficiently inhibit the tube-like structure formation by about 50% when added at 200 ng/ml (Figures 2Bc, 2Bd, lower panel). On the contrary, addition of different TIMPs to EPC culture medium did not impair tube-like structure network on Matrigel® (Figure 2C). These data suggested that MT-MMPs were involved in the regulation of tube-like structure formation in MSC.

### Lumican Preferentially Down-regulates MMP-14 Expression and Activity in MSC

**Figure 3:**
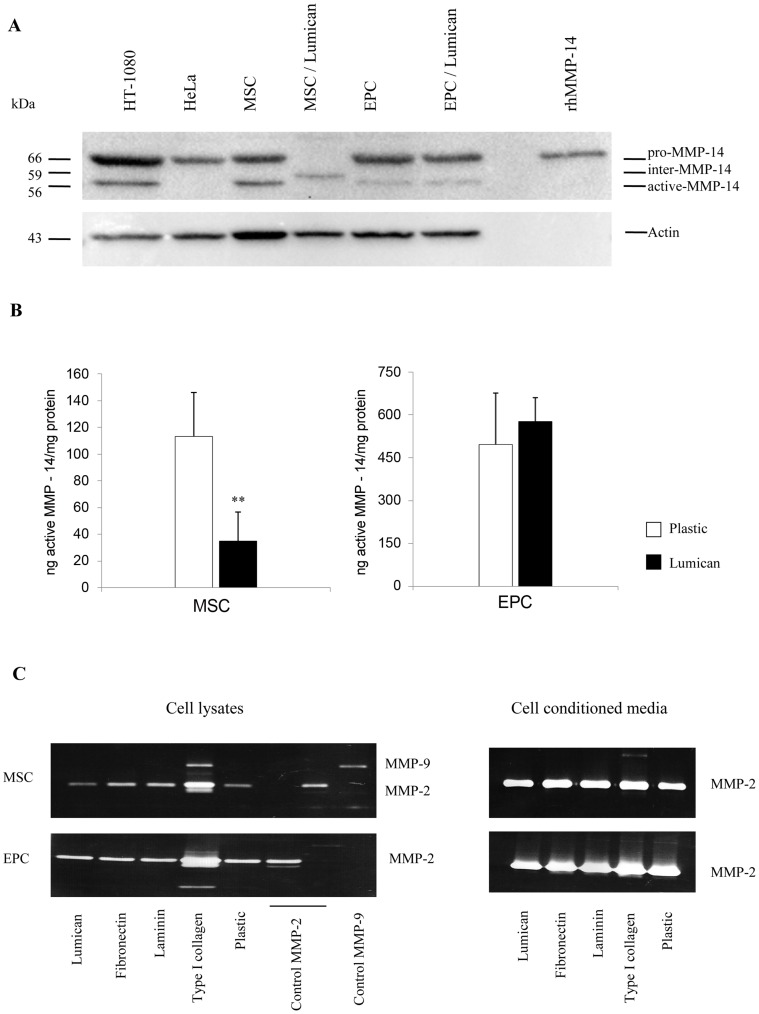
Lumican inhibits MMP-14 expression and activity in MSC. (A): Lumican effect on MMP-14 expression in MSC and EPC analyzed by Western blotting using a mouse anti-MMP-14 monoclonal antibody directed against the catalytic domain of MMP-14 and probing with anti-β-actin. Semi-confluent MSC and EPC were seeded on plastic or in presence of lumican coating as described in Materials and Methods section. Inactive and active MMP-14 forms were detected in positive control cell lysates of fibrosarcoma cell line (HT-1080). Active MMP-14 was not detected in negative control cell lysates of HeLa cells. (B): MMP activity was measured using a SensoLyte® 520 MMP-14 Assay Kit as described in the Materials and Methods section. The activity of MMP-14 of cell lysates of MSC and EPC seeded on plastic or lumican coating (30 µg/cm^2^) was measured from four different donors in two independent experiments. Data presented as mean ± S.D. **p<0.01. (C): MMP-2 and MMP-9 expression and activity in MSC and EPC. MMP-2 and MMP-9 expression and activity in cell lysates (left panel) and conditioned cell media (right panel) were analyzed by zymography.

Previous studies showed that MMP-14 is particularly involved in lumican-dependent inhibition of *in vitro* and *in vivo* angiogenesis [30]. So, MMP-14 expression in our cells was analyzed by Western blotting (Figure 3A). Using a mouse anti-MMP-14 monoclonal antibody directed against the catalytic domain of MMP-14 (Figure 3A), inactive (∼66 kDa) and active (∼56 kDa) forms of MMP-14 were detected in HT-1080 (positive control), in MSC and EPC seeded on plastic. The inactive (∼66 kDa) form was detected in HeLa cells and the recombinant human MMP-14 sample. When seeded on lumican for seven days, MSC expressed an intermediate (∼59 kDa) form of MMP-14 while inactive (∼66 kDa) and active (∼56 kDa) forms of MMP-14 were not detected. In contrast, EPC expressed similar inactive (∼66 kDa) and active (∼56 kDa) forms of MMP-14 when seeded on lumican. In addition, Western blotting analysis gave evidence of efficient down-regulation of MMP-14 protein expression in MSC seeded on lumican coating, compared to the other ECM protein substrata (not shown). In contrast to MSC, the level of both MMP-14 forms in EPC was not changed when seeded on lumican, compared to those growing on fibronectin and plastic. A slight up-regulation was observed with type I collagen and laminin only (not shown).

Subsequently, MSC and EPC seeded on lumican or plastic were collected and tested for MMP-14 activity (Figure 3B). When seeded on lumican, MSC exhibited an inhibition of MMP-14 activity, compared to control cells cultured on plastic. MMP-14 activity was not influenced by lumican in EPC in the same assay.

The formation of tube-like structures by MSC and endothelial cells was reported to be regulated by MMP-2, -9 and their inhibitors [31]. Hence, expression and activity of both gelatinases were analyzed in cells cultured on various ECM protein coatings (lumican, type I collagen, fibronectin or laminin) and MMP secretion profiles were analyzed by gelatin zymography (Figure 3C). EPC exhibited higher level of pro-MMP-2 expression than MSC (Figure 3C, left panel). In addition, in both cell types, the appearance of processed forms (intermediate and active) of MMP-2 could be observed in the presence of type I collagen. MMP-9 was detected in MSC seeded on type I collagen only (Figure 3C, left panel). In parallel to the cell lysates, relevant conditioned media were also collected and assayed for MMP-2 and MMP-9 activities. MSC and EPC secreted similar level of proMMP-2 (Figure 3C, right panel).

Using real-time PCR, a set of human MMP (MMP-1, -2, -9, -13, -14, -15, -16) and TIMP genes (supplemental material, Table S2) was explored to broadly establish the MMP expression profile of MSC cultured either on plastic or lumican coating. During *in vitro* culture on uncoated plastic, MSC expressed MMP-1, -2, -14, -16 and TIMP-1, -2, -3 mRNAs, whereas MMP-9, -13, -15 and TIMP-4 mRNAs were not detected under these conditions. Importantly, the levels of every MMP transcripts were found to be similar between cells growing on plastic and lumican-coated plates (not shown).

Taken together, these results showed that lumican down-regulated MMP-14 expression and activity in MSC in a preferential manner, and that most of the regulation took place at the post-transcriptional level.

### Lumican Inhibits MSC Directional Migration and Invasion

**Figure 4:**
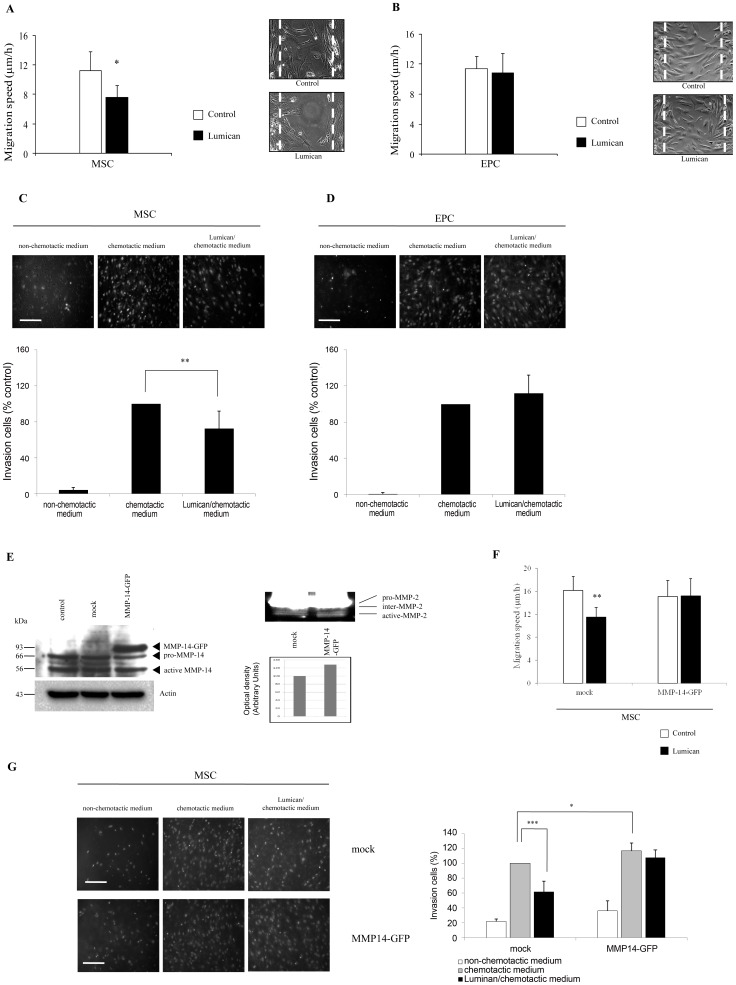
Lumican inhibits MSC migration and invasion. MSC (A) or EPC (B) were plated on 12-well plates at 15 000 cells per chamber of culture-insert. Cells were incubated at 37°C and 5% CO_2_ for 24 h. After withdrawal of the culture-insert, lumican (100 nM) was added to the serum-free cell culture medium. The migration speed of MSC and EPC was determined by means of computer-assisted phase contrast videomicroscopy during 48 hours as described in Materials and Methods. Representative images of cell positions after 48 h of migration are displayed on the right panels. The data are representative of three independent analyses. *p<0.05. (C) MSC and EPC (D) invasion in presence of lumican. Cells were seeded on inserts as described in the Materials and Methods section. Medium with 10% or 2% FBS was used as a chemotactic agent for MSC or EPC, respectively, and the cells were cultured for a further 48 h. Negative control medium contained 2% BSA. Medium containing 0.5% BSA and 100 nM lumican was added to the upper chamber at the time of seeding. Invading cell nuclei were stained with Hoechst 33342 (5 µg/mL) and counted using fluorescence microscopy. Representative fluorescence images of invading cells are displayed on the upper panel. Results are expressed as the percentage of control (cells invading toward chemotactic medium) (mean ± S.D.) from at least three independent experiments with triplicate inserts. Scale bar: 100 µm. **p<0.01; (E, upper panel): Lysates from transiently transfected MSC with GFP (mock) or MMP-14-GFP constructs were analyzed for MMP-14 overexpression by Western blotting with an antibody raised against MMP-14 after 48 h of transfection. Non-transfected MSC lysate is also included as control. (E, bottom panel): Overexpression of MMP-14 in MSC induces MMP-14 activity as shown by the enhancement of active MMP-2 band by zymography. (F): Migration of GFP- (mock) or MMP-14-GFP-transfected MSC was determined 48 h post-transfection using cell culture-insert assay. The migration speed of GFP-positive MSC, was determined by means of computer-assisted phase contrast and fluorescence videomicroscopy during 24 hours as described in supplemental methods section. The data are representative of two independent analyses. **p<0.01. (G): GFP- (mock) or MMP-14-GFP-transfected MSC invasion in presence of lumican. Cells were cultured and inserts seeded as described in the supplemental methods section. Medium with 10% FBS was used as a chemotactic agent for MSC, and the cells were cultured for a further 48 h. Negative control medium contained 2% BSA. Medium containing 0.5% BSA and 100 nM lumican was added to the upper chamber at the time of seeding. Invading cell nuclei were stained with Hoechst 33342 (5 µg/mL) and counted using fluorescence microscopy. Representative fluorescence images of invading cells are displayed on the upper panel. Results are expressed as the percentage of control (mock-transfected cells invading toward chemotactic medium) (mean ± S.D.) from two independent experiments with triplicate inserts. Scale bar: 100 µm. *p<0.05. ***p<0.001.

Since stem cell migration is crucial for their biological properties, lumican effect on MSC (Figure 4A) and EPC (Figure 4B) migration was analyzed by an *in vitro* wound healing assay. In the control dishes, MSC completely filled the wound after 48 h. In contrast, addition of 100 nM lumican in the serum-free cell culture medium decreased the MSC migration (Figure 4A). The migration speed of MSC was 11.2±2.6 µm/h in the controls *versus* 7.6±1.7 µm/h in the presence of 100 nM lumican. Thus, the presence of 100 nM lumican significantly decreased the MSC migration speed by nearly 30% as compared to control. In contrast to MSC, the presence of lumican did not impair the EPC migration speed (11.4±1.6 µm/h in the controls *versus* 10.8±2.6 µm/h in the presence of lumican) (Figure 4B).

In addition, MSC migration assays were performed with 10 µg/ml blocking antibodies raised against human α2 and β1 integrin subunits in presence or absence of lumican (100 nM) (supplemental material, Figure S6). After 24 h of incubation in presence of lumican but in absence of blocking antibodies, the migration speed of MSC (12.7±1.2 µm/h) was significantly decreased as compared to control (16.4±0.8 µm/h), as expected (supplemental material Figure S6), (*p*<0.001).

After 24 h of incubation in absence of lumican, but in presence of blocking antibodies, the blocking antibody raised against human β1 integrin did not alter significantly the migration speed of MSC (16.0±1.3 µm/h versus 16.4±0.8 µm/h), (supplemental material, Figure S6). Therefore, the blocking antibody raised against human β1 integrin alone was not sufficient to inhibit MSC migration. In presence of lumican, the blocking antibody raised against human β1 integrin abolished the inhibitory effect of lumican on MSC migration speed (15.5±0.6 µm/h *versus* 12.7±1.2 µm/h, *p*<0.01).

After 24 h of incubation in absence of lumican, the blocking antibody raised against human α2 integrin decreased slightly but significantly the migration speed of MSC (14.0±0.9 µm/h) as compared to MSC incubated without blocking antibody (16.4±0.8 µm/h, *p*<0.05), (Figure S6). Therefore, the blocking antibody raised against α2 integrin alone was able to inhibit MSC migration. In presence of lumican, the blocking antibody raised against human α2 integrin more strongly decreased the migration speed of MSC (12.5±0.7 µm/h, *p*<0.01). However, in this condition, the inhibition of MSC migration speed was similar to the inhibition induced by lumican alone. Therefore, no synergistic effect of lumican and α2 blocking antibody could be observed. These results suggest that α2 integrin might regulate MSC migration but is not implicated in the lumican-induced decrease of MSC migration speed (supplemental material, Figure S6).

We next investigated whether lumican could prevent MSC and EPC invasion through reconstituted basement membrane, Matrigel® (Figures 4C, 4D). Our results showed that after 48 h incubation in the presence of 100 nM lumican in the culture medium, the number of cells passed through the pre-coated inserts was significantly decreased (−30%), compared to the control without lumican (Figure 4C). Using EPC in the same assay, we found that these cells were also able to invade the Matrigel® gels. However, again, we did not observe any significant effect of lumican on EPC (Figure 4D). Collectively, these data showed that lumican was capable of hindering MSC invasion triggered by chemotactic agent.

### Lumican-specific Impairment in MSC Migration and Invasion can be Restored by MMP-14 Overexpression

In view of the above observations, a gain-of-function assay was performed to estimate if MMP-14 overexpression would reverse the migration defect of MSC. As shown in Figure 4E (upper panel), MMP-14-GFP fusion protein could be detected after 48 h of transfection by Western blotting probed with an anti-MMP-14 antibody. Overexpression of MMP-14 in MSC caused enhancement (+30%) of active MMP-2 fraction in gelatin zymography (Figure 4E, bottom panel). Subsequent cell culture-insert migration assay showed an increase of migration speed of MMP-14-GFP-transfected MSC in comparison to non-transfected cells even in the presence of 100 nM lumican as early as after 24 h (Figure 4F). By contrast, MSC transfected with GFP only still exhibited significant migration defect when cultured with lumican (16.14±2.45 µm/h *vs* 11.51±1.70 µm/h).

MMP-14 overexpression in MSC led to reconstitute the invasive potential of MSC, regardless of the presence of lumican (Figure 4G). Conversely, mock-transfected MSC still exhibited defective invasion characteristics (decreased by 40%) in the presence of 100 nM lumican, as the non-transfected MSC.

Taken together, these results confirmed that inhibition of MSC migration and invasion by lumican was abolished by MMP-14 overexpression and that MMP-14 was a key mediator during these processes.

## Discussion

In order to migrate at distal sites of the bone marrow environment, MSC induce specific MMP activity, which is mediated by chemokines and cytokines involved in the regulation of the immune or inflammatory process [32]. The involvement of EPC in neovascularization is due to their capacity to contribute to tumor angiogenesis *in vivo*. MSC may also contribute to a vascular niche by providing growth factors rather than by incorporating into vascular structures. Bone marrow MSC can provide a local environment that favours migration and vascularisation of the surroundings of injured sites [9]. Since lumican was identified recently as a potential regulator of angiogenesis [23], [30], the aim of the present study was to analyze whether lumican had an effect on precursor vascular cells such as EPC and MSC. Our results indicate that lumican inhibited migration and tube-like structure formation by MSC *via* reducing both MMP-14 expression and activity.

Using flow cytometry, specific markers of MSC like CD73 and CD90 were detected in MSC (Figure 1A). In contrast, endothelial cell specific marker (vWF) was only detected in EPC by flow cytometry, as already shown [33]. EPC expressed intermediate level of CD31 between MSC and HUVEC (Figure 1C) demonstrating the immature endothelial phenotype of EPC. In addition, transcriptomic analysis was performed because it was thought to provide a useful tool for characterization of MSC [34]. Among 63 genes differently expressed in MSC and EPC, several genes well described in MSC were down-regulated in EPC, including stromal cell derived factor-1 (SDF-1) [35], and some genes that contribute to the formation of the extracellular matrix, like versican [34], or Cartilage Oligomeric Matrix Protein (COMP). Interestingly, secreted frizzeled-related protein 4, which belongs to a receptor family binding Wnt and described to control the fate of MSC [36], was also significantly down-regulated in EPC. Among the top 12 molecules which were up-regulated in EPC as compared to MSC, apolipoprotein D expressed in perivascular cells and pericytes, and carboxypeptidase M which cleaves SDF-1 were reported to regulate MSC cell recruitment (Table S1) [35]. Altogether, these microarray data, allowed us to validate the *in vitro* differentiation protocol.

The bone marrow ECM is highly organized and is composed of type I, III, IV, V and VI collagens, fibronectin, laminin, various proteoglycans such as perlecan, and members of the SLRP family including biglycan and decorin [37]. Although it is well established that bone marrow ECM components, like biglycan and decorin, play a critical role in the differentiation of MSC [11], little is known about the influence of lumican, another SLRP member, on this biological phenomenon.

In our study, cells were differentiated into EPC for 13 days with regular changes of the VEGF-supplemented basal medium [6], [38]. In contrast to the results reported by Oswald and collaborators [5], a seven day treatment by 50 ng/ml VEGF was not sufficient to induce EPC phenotype and a CD31 or vWF positive staining. Similar result to our study was also reported by other groups [39]. As compared to glass non-coated coverslips, the presence of ECM proteins had no direct effect on EPC differentiation [31]. We demonstrated that the presence of lumican did not inhibit VEGF-induced differentiation of MSC to EPC phenotype.

Lumican was reported to be pro-apoptotic by modulating Fas-Fas ligand signaling in cornea [40], and in blood vessels [26]. We showed that lumican had no effect either on MSC or EPC proliferation, or apoptosis (Figure S3). In our case, Fas receptor was more expressed in MSC than in EPC. The down-regulation of Fas receptor by VEGF-induced endothelial cells was already reported [41].

In this study, we demonstrated that exogenous lumican effect is clearly cell-type specific. To understand this differential effect, we studied the endogenous lumican expression profile in both cell types, MSC and EPC (data not shown). We observed that expression of lumican (57 kDa) increased with the degree of differentiation towards an endothelial cell phenotype. These data suggest that MSC might respond better than EPC to exogenous lumican. In other words, stronger expression of endogenous lumican in endothelial cells might impair their responsiveness to exogenous lumican. The level of active MMP-14 in MSC and EPC might also explain the different responses of these cells to lumican. MMP-14 activity is far higher in EPC than in MSC (see Figure 3B), which can explain their resistance to lumican inhibition.

Interestingly, α2 integrin subunit, to which lumican binds directly [22], was detected at a similar level of expression in MSC and EPC (Figure 1D) and as such, differences of α2 integrin expression could not explain the difference of response to lumican of these two cell types. The higher expression of β1 integrin subunit in MSC as compared to EPC might suggest a role of β1 integrin subunit in the lumican inhibition of MSC migration.

Lumican might inhibit MSC migration *via* β1 integrin, as suggested by the abolishing effect of the anti-human β1 integrin antibody on lumican inhibition of MSC migration (Supplemental Material, Figure S6). However, the absence of effect of lumican on the actin cytoskeleton organization (Supplemental Material, Figure S1) and on the FAK-pY397/Total FAK ratio in MSC (Supplemental Material, Figure S2) does not support this hypothesis. Lumican rather increased the cell adhesion *via* β1 integrin as we already published [21]. Our present results also suggest that β2 integrin might regulate MSC migration but the anti-human α2 integrin blocking antibody did not abolish the lumican inhibitory effect on MSC migration (Supplemental Material, Figure S6). These results suggest that the lumican-induced inhibition of migration of MSC is regulated by a mechanism which poorly involves β2 integrin.

Consequently, we suggest that the inhibiting effect of lumican on MSC migration might be explained mainly by its inhibitory effect on MMP-14 expression and activity. Lumican may act directly on MMP-14 activation independently of α2β1 integrin. Since β2 integrin has been shown to interact with lumican in circulating cells [42], a perspective of our work would be to analyze the role of β2 integrin in the regulation of lumican inhibition of MSC migration.

MSC and EPC are able to form tube–like structures on Matrigel® [31], [43], [44]. The capacity to form such tubular structures *in vitro* is not by its own sufficient to define an “angiogenic property”. However, this *in vitro* assay of vasculogenesis is commonly used for EPC and MSC, even if it only refers to one step of a complex process.

Bone marrow MSC can provide a local environment that favours migration and vascularisation of the surroundings of injured sites [9]. Moreover, when co-cultured, MSC participate to tube-like formation in a very similar way to EPC [44]. In the context of tissue regeneration, vascular support provided by MSC is a beneficial feature, able to accelerate the healing of injured sites within the body. On the other side, this characteristic makes these cells attracted to tumors, where they may support angiogenesis and facilitate tumor growth and metastasis. Studies have shown that up to 90% of endothelial cells in early tumors originate from bone marrow-derived stem cells [27]. Surprisingly, in the presence of lumican, pseudotube formation by EPC was not altered, while MSC did not create pseudotubular network (Figure 2A). However, as EPC are also able to migrate and invade Matrigel® in the presence of lumican, we conclude that, once differentiated, these specialized cells lose, at least partially, their capacity to respond to ECM signals while preserving the potential to differentiate into endothelial cells. MSC and mature endothelial cells respond to lumican, which suggests that endothelial progenitors may represent an intermediate stage and may be considered as the cells remaining in a kind of physiological “dormancy”. Lumican inhibition of tube-like formation by MSC, but not by dermal fibroblasts or EPC, does not simply rely on a difference of origin, mesenchymal *versus* endothelial (supplemental material, Figure S5). The absence of effect of exogenous lumican on dermal fibroblasts might be explained by the high endogenous level of expression of lumican by these cells.

Bone marrow stem cell niche is a particular microenvironment where ECM is a functional component. It consists of various proteins, including lumican [45]. Our data suggest that lumican, which considerably decreases the migration and the invasion of MSC, may be viewed as an effective factor retaining stem cells in their niche.

Migration and invasion of MSC are characterized by the production of enzymes that cause proteolytic modification of proteins in the ECM. Such MSC mobilization requires coordinated action of MMPs and their inhibitors, such as TIMPs [31], [39]. EPC are immature endothelial cells. Therefore, their response to TIMPs may be not identical to that of mature cells. We demonstrated that the level of MMP-14 activity is weaker in MSC than in EPC as shown in figure 3B. This may explain the different responses of these cells to Gallardin® (Figure S4). The difference of MMP-14 activity in MSC and EPC may also explain their difference of response to TIMPs (Figure 2). Lumican was shown to interact with type I collagen, masking cleavage sites of MMP-1 and MMP-13 [46], two proteases described to regulate MSC migration [47], [48]. Since lumican did not alter MMP-2 activity (Figure 3C) and since the MMP-1, -2, -14, -16 transcript levels were also found to be similar in presence of lumican, we focused on the study of the effects of lumican on the protein expression and activity of MMP-14, due to the critical role of this MMP in the control of cell migration and invasion. It was reported that MMP-14 regulates the migration of mature endothelial cell [49], but also of MSC [2], [31] and EPC [31]. Recently, Niewiarowska *et al*. reported that lumican can down-regulate MMP-14 by interfering with α2β1 integrin in experimental angiogenesis [30]. Herein, we showed that lumican inhibited MMP-14 expression and activation in MSC only and not in EPC (Figure 3A), as compared to other ECM substrata. The induction of an intermediate form of MMP-14 suggests an abnormal maturation or trafficking of this MMP. The mechanism of lumican inhibition on MMP-14 expression will require further analyses.

The MMP-14 activity was decreased in MSC, but not in EPC, when cells were cultured on lumican (Figure 3B). These results suggest that lumican may inhibit cell migration of MSC *via* an inhibition of MMP-14 expression and activity. On the other hand, lumican (and decorin) can be degraded by MMP-14, abrogating the anti-tumour effect of these proteins [50], [51]. Furthermore, as the inhibiting effect of lumican on MSC migration and invasion was suppressed by overexpression of MMP-14 (Figures 4F, 4G), we conclude that lumican regulation of MSC migratory and invasive abilities are MMP-14-dependent. It was observed that MSC were able to initiate an invasion process that was regulated by MMP-14 [2].

Altogether, our data show that lumican inhibits MSC migration and invasion through a decrease of MMP-14 expression and activity. The lumican inhibitory effect was MSC-specific since EPC migration and tubulogenesis were not impaired. We suggest that lumican could down-regulate the MSC migration and invasion with MMP-14 as the main target. Our report highlights the important role of lumican in the local environment of MSC, modulating their biological functions. A better understanding of the interactions that occur between ECM and MSC dynamics could lead to more effective stem cell-based therapies.

## Supporting Information

Figure S1 **Actin cytoskeleton distribution in MSC and EPC seeded on non-coated glass coverslips or coated with type I collagen, fibronectin, and lumican.** MSC (a–d) and EPC (e–h) were grown to 80% confluence for 24 h on glass coverslips (a, e) pre-coated with 30 µg/cm^2^ type I collagen (b, f), 10 µg/ml fibronectin (c, g), or 30 µg/cm^2^ lumican (d, h). The distribution of actin cytoskeleton was not significantly altered in MSC and EPC in presence of lumican as compared to other ECM substrata. Scale bar: 20 µm.
(TIF)

Figure S2 **Expression of FAK-pY397 and total FAK in MSC and EPC.** Cells were grown to confluence on 6-well plate. Expression of FAK-pY397 and total FAK in MSC and EPC was analyzed by Western immunoblotting after 15 min incubation without or with 100 nM of lumican. The levels of FAK-pY397 and total FAK were quantified by densitometric analysis and the FAK-pY397/FAK ratios were determined.
(TIF)

Figure S3 **Effect of lumican on the proliferation and the apoptosis of MSC and EPC.** (A): Proliferation assay of MSC and EPC cultured on plastic or lumican-coated (30 µg/cm^2^) 6-well plates for 7 or 21 days, respectively. The results were reported as mean values (O.D. 560) ±S.D. (n = 3). (B): Cells were grown to 80% confluence for 24 h on coverslips pre-coated with type I collagen (30 µg/cm^2^), fibronectin (10 µg/ml), or lumican (30 µg/cm^2^). The cell cultures were stained with Hoechst 33342. Scale bar: 20 µm. (C): Semi-confluent cells were maintained for 7 days (MSC) or 21 days (EPC), in presence of different ECM coatings: type I collagen (30 µg/cm^2^), fibronectin (10 µg/ml), recombinant human lumican (30 µg/cm^2^). Cyclin D1, Bax, Bcl-2, FasR, and actin protein expression in MSC and EPC was then analyzed by Western blotting as described in Materials and Methods. (D): The levels of Bax and Bcl-2 were quantified by densitometric analysis and Bax/Bcl-2 ratios were determined. These results are representative of three independent experiments. Data are expressed as means ± S.D.
(TIF)

Figure S4 **Gallardin® effect on MSC and EPC tube-like formation.** Tube formation on Matrigel® (a-d) in control (a, c) or 10^−9^ M Gallardin® supplemented medium (b, d) was observed 24 hours after MSC (a, b) and EPC (c, d) seeding. Representative photographs are presented on the left panel. The semi-quantitative evaluation of the tube network from ten randomly selected fields was performed using ImageJ software and NeuronJ plugin (right diagrams). Experiments were performed in triplicate on three different donors. Results represent the mean ± S.D. Scale bar: 180 µm *p<0.05.
(TIF)

Figure S5 **Lumican effect on dermal fibroblast tube-like formation.** Tube formation on Matrigel® in control (a) or 100 nM lumican supplemented medium (b) was observed 24 hours after dermal fibroblast seeding (a, b). Representative photographs are presented on the left panel. The semi-quantitative evaluation of the tube network from ten randomly selected fields was performed as described above (right diagrams). Experiments were performed in triplicate on three different donors. Results represent the mean ± S.D. Scale bar: 180 µm.
(TIF)

Figure S6 **Effect of blocking antibodies raised against human β1 and α2 integrin subunits on MSC migration in presence or absence of lumican.** Blocking antibodies (10 µg/ml) anti-human α2 (MAB 1950) and β1 (MAB 1951) integrin subunits were incubated with MSC during 24 h *in vitro* wound healing assays in presence or absence of 100 nM lumican. The cell migration was video-recorded and the migration speed was measured as described in the materials and methods section. The results are representative of the two independent experiments. Data are expressed as means ± SD. (*p<0.05, **p<0.01, ***p<0.001).
(TIF)

Material S1 **Supplemental Materials.**
(DOC)

Table S1 **Top 12 genes down-regulated and up-regulated in EPC** ***versus*** **MSC.**
(DOC)

Table S2 **List of primers used for quantitative real time PCR reaction.**
(DOC)
