# Supplementary figures and images for: Effect of Lumican on the Migration of Human Mesenchymal Stem Cells and Endothelial Progenitor Cells: Involvement of Matrix Metalloproteinase-14
Source: PLoS One. 2012 Dec 7;7(12):e50709. doi: 10.1371/journal.pone.0050709 (PMC3517548; doi:10.1371/journal.pone.0050709)

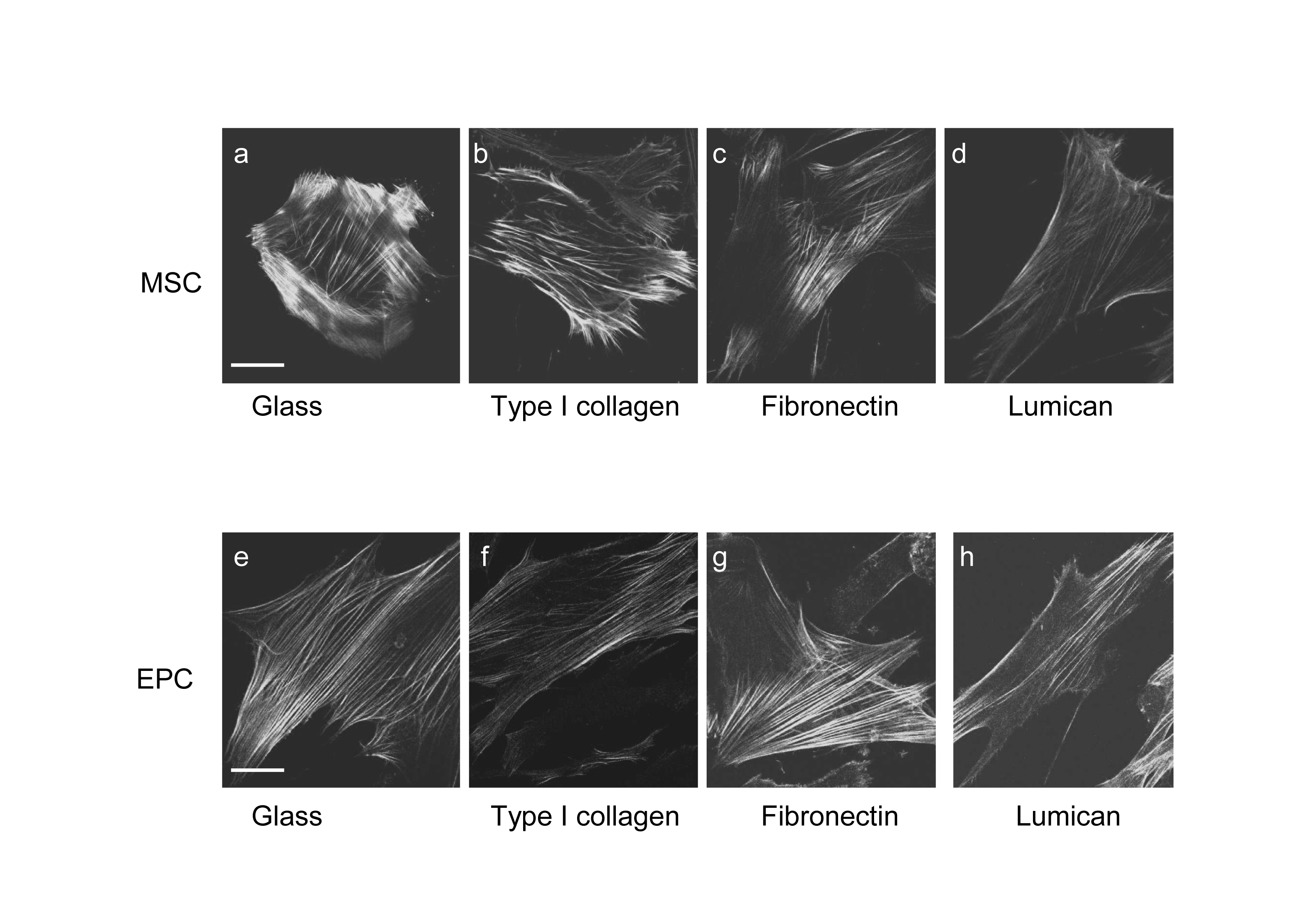

Supplement: Figure S1 — Actin cytoskeleton distribution in MSC and EPC seeded on non-coated glass coverslips or coated with type I collagen, fibronectin, and lumican. MSC (a–d) and EPC (e–h) were grown to 80% confluence for 24 h on glass coverslips (a, e) pre-coated with 30 µg/cm2 type I collagen (b, f), 10 µg/ml fibronectin (c, g), or 30 µg/cm2 lumican (d, h). The distribution of actin cytoskeleton was not significantly altered in MSC and EPC in presence of lumican as compared to other ECM substrata. Scale bar: 20 µm. (TIF) [file pone.0050709.s001.tif]

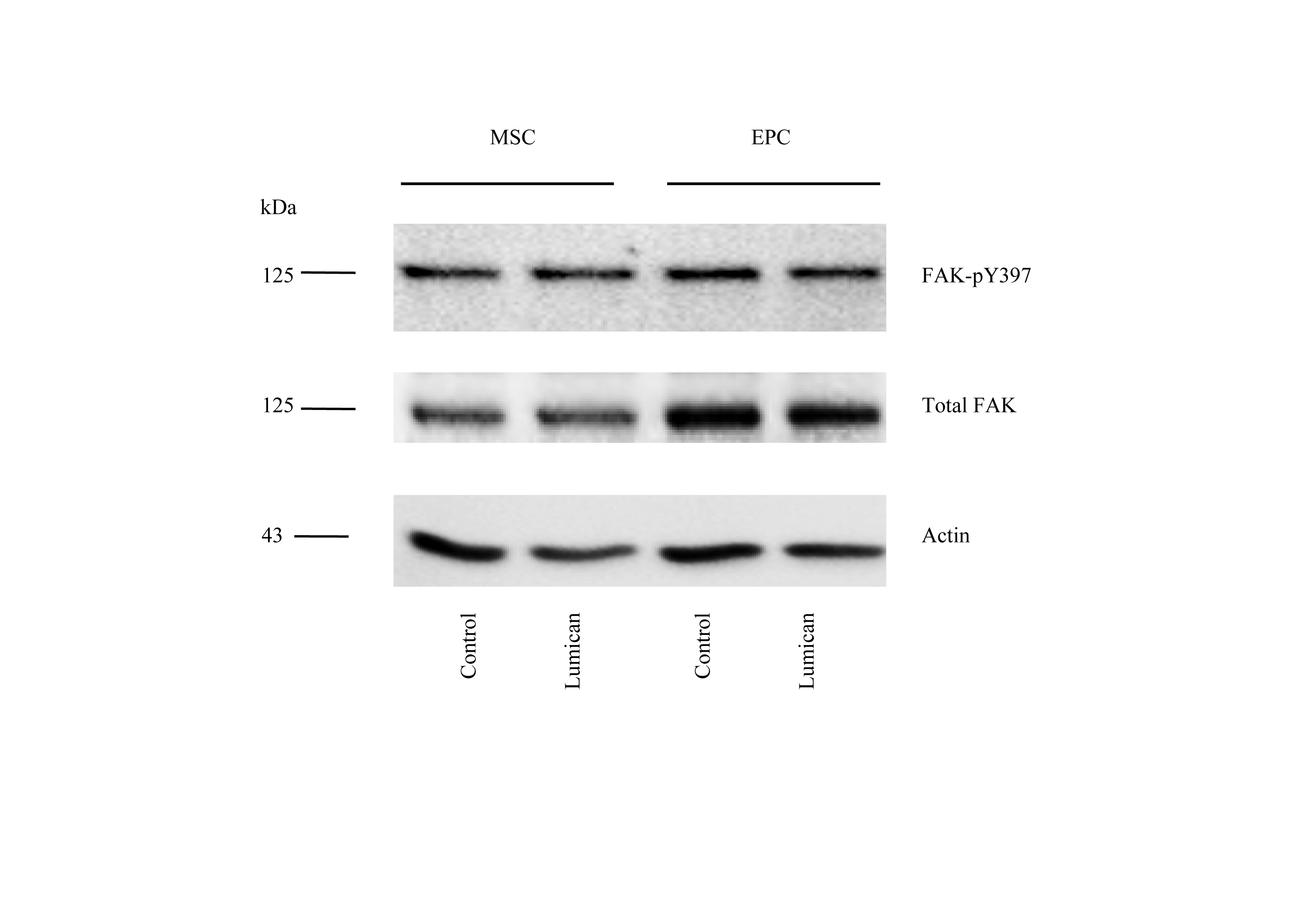

Supplement: Figure S2 — Expression of FAK-pY397 and total FAK in MSC and EPC. Cells were grown to confluence on 6-well plate. Expression of FAK-pY397 and total FAK in MSC and EPC was analyzed by Western immunoblotting after 15 min incubation without or with 100 nM of lumican. The levels of FAK-pY397 and total FAK were quantified by densitometric analysis and the FAK-pY397/FAK ratios were determined. (TIF) [file pone.0050709.s002.tif]

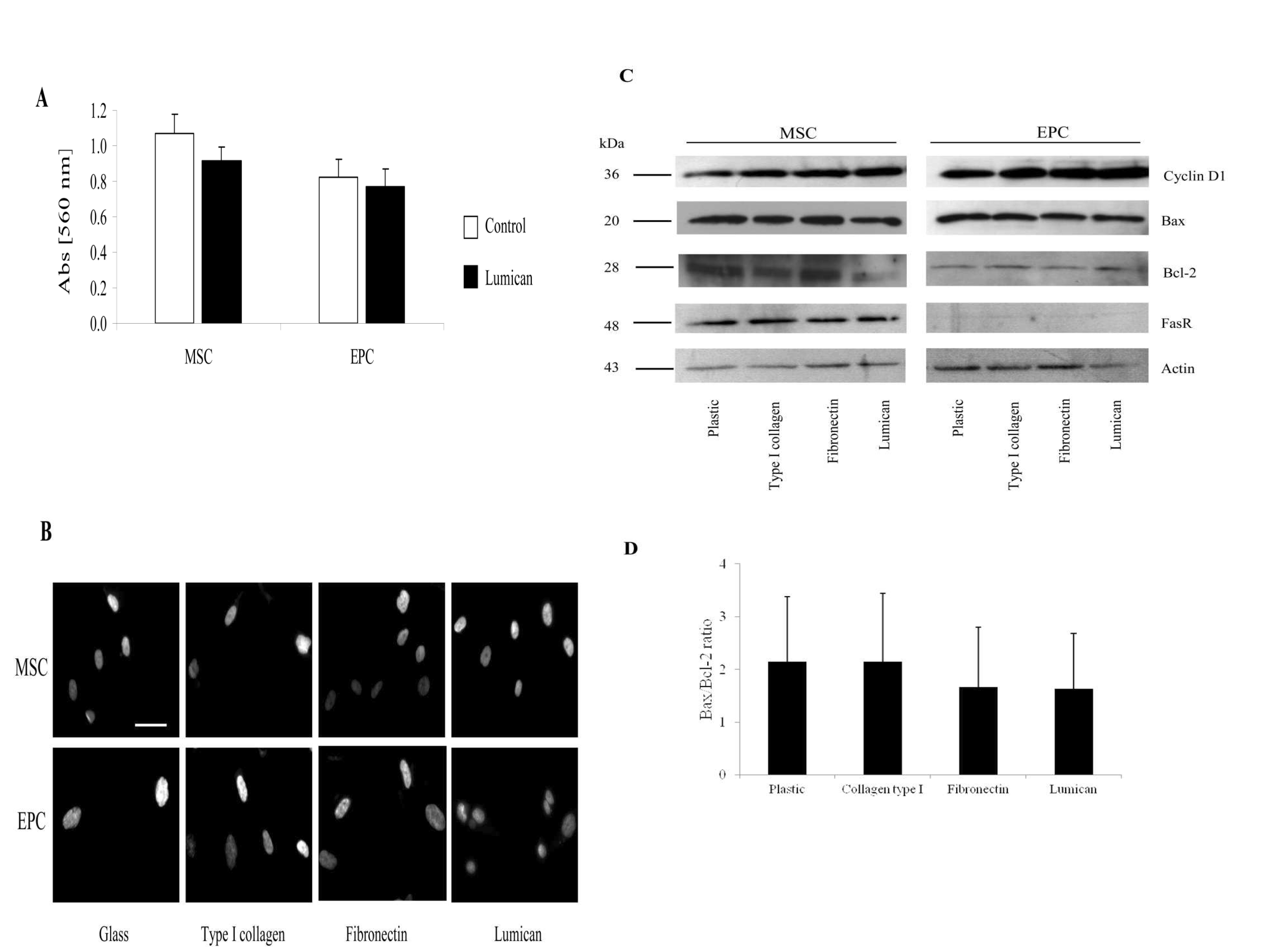

Supplement: Figure S3 — Effect of lumican on the proliferation and the apoptosis of MSC and EPC. (A): Proliferation assay of MSC and EPC cultured on plastic or lumican-coated (30 µg/cm2) 6-well plates for 7 or 21 days, respectively. The results were reported as mean values (O.D. 560) ±S.D. (n = 3). (B): Cells were grown to 80% confluence for 24 h on coverslips pre-coated with type I collagen (30 µg/cm2), fibronectin (10 µg/ml), or lumican (30 µg/cm2). The cell cultures were stained with Hoechst 33342. Scale bar: 20 µm. (C): Semi-confluent cells were maintained for 7 days (MSC) or 21 days (EPC), in presence of different ECM coatings: type I collagen (30 µg/cm2), fibronectin (10 µg/ml), recombinant human lumican (30 µg/cm2). Cyclin D1, Bax, Bcl-2, FasR, and actin protein expression in MSC and EPC was then analyzed by Western blotting as described in Materials and Methods. (D): The levels of Bax and Bcl-2 were quantified by densitometric analysis and Bax/Bcl-2 ratios were determined. These results are representative of three independent experiments. Data are expressed as means ± S.D. (TIF) [file pone.0050709.s003.tif]

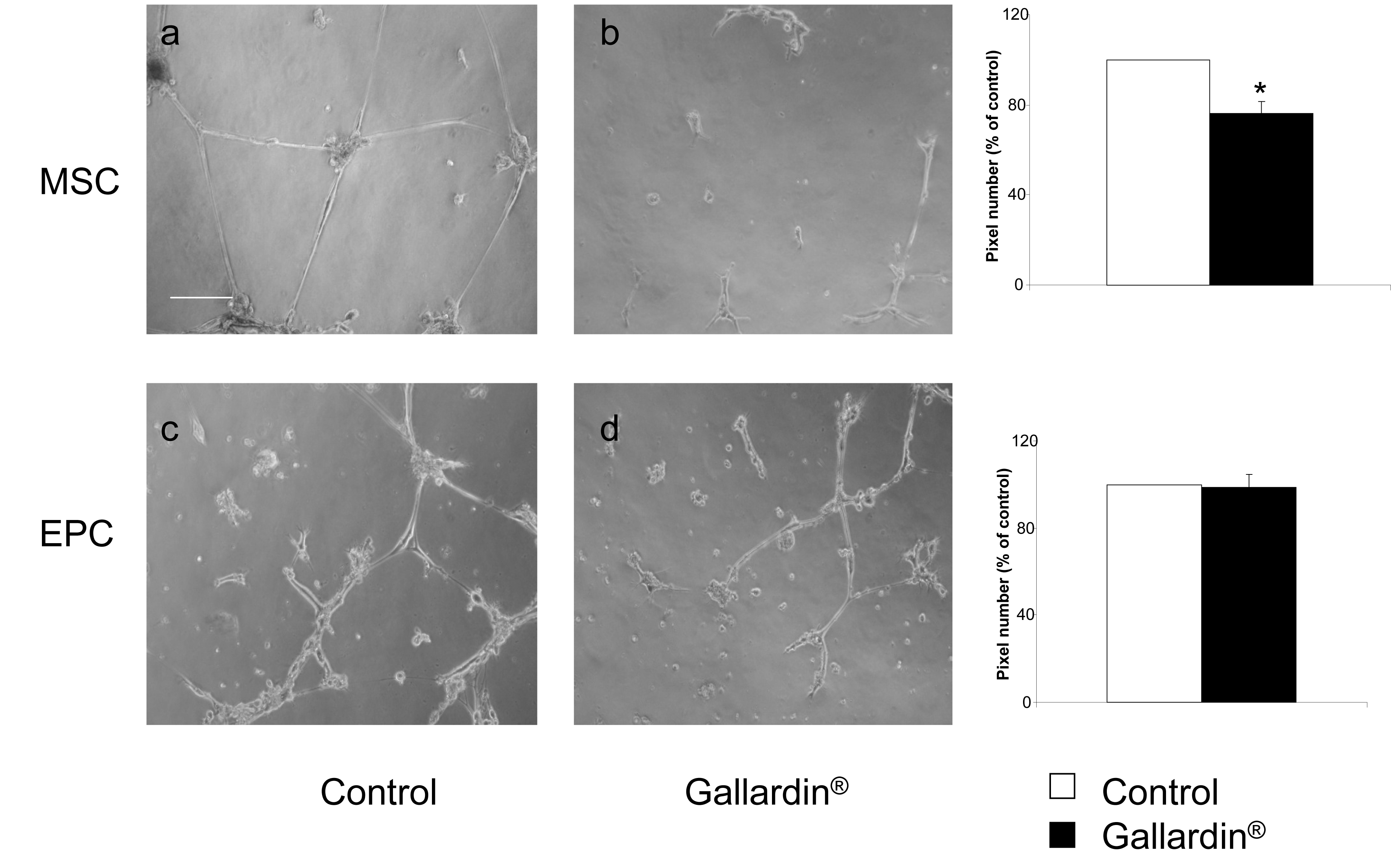

Supplement: Figure S4 — Gallardin® effect on MSC and EPC tube-like formation. Tube formation on Matrigel® (a-d) in control (a, c) or 10−9 M Gallardin® supplemented medium (b, d) was observed 24 hours after MSC (a, b) and EPC (c, d) seeding. Representative photographs are presented on the left panel. The semi-quantitative evaluation of the tube network from ten randomly selected fields was performed using ImageJ software and NeuronJ plugin (right diagrams). Experiments were performed in triplicate on three different donors. Results represent the mean ± S.D. Scale bar: 180 µm *p<0.05. (TIF) [file pone.0050709.s004.tif]

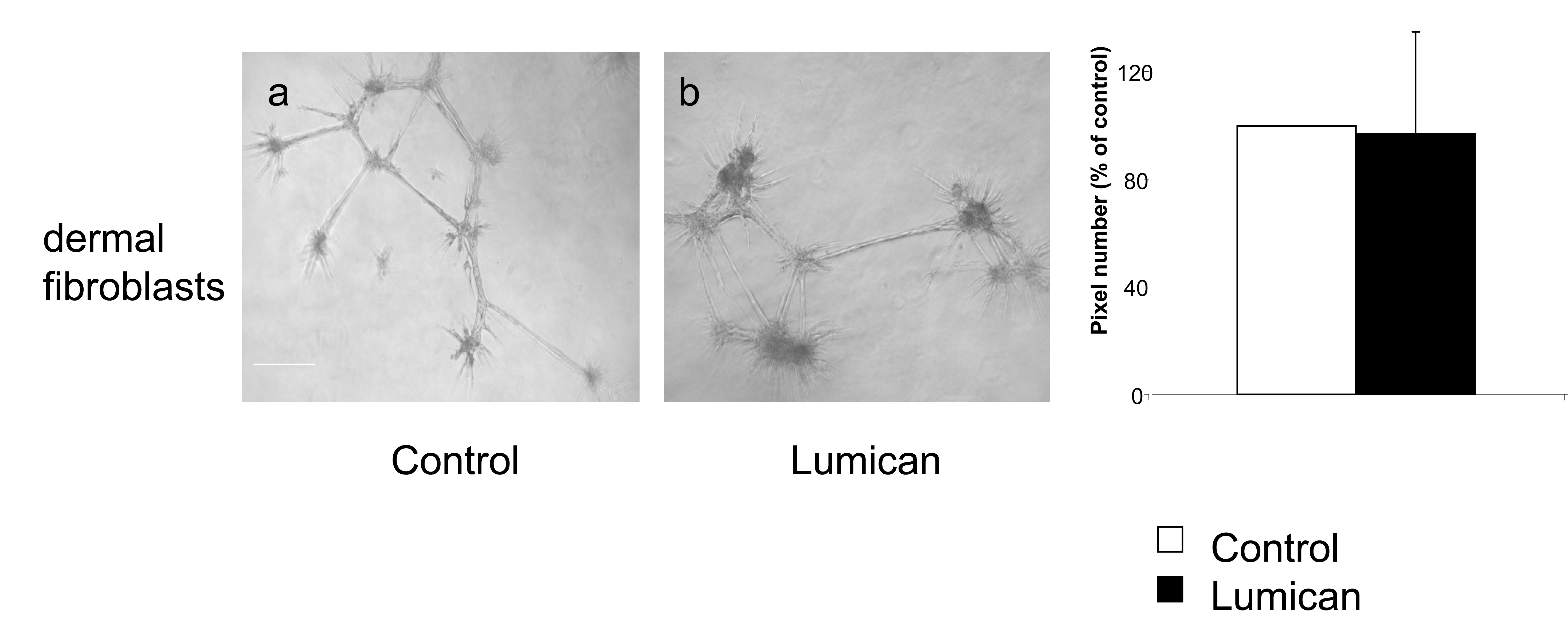

Supplement: Figure S5 — Lumican effect on dermal fibroblast tube-like formation. Tube formation on Matrigel® in control (a) or 100 nM lumican supplemented medium (b) was observed 24 hours after dermal fibroblast seeding (a, b). Representative photographs are presented on the left panel. The semi-quantitative evaluation of the tube network from ten randomly selected fields was performed as described above (right diagrams). Experiments were performed in triplicate on three different donors. Results represent the mean ± S.D. Scale bar: 180 µm. (TIF) [file pone.0050709.s005.tif]

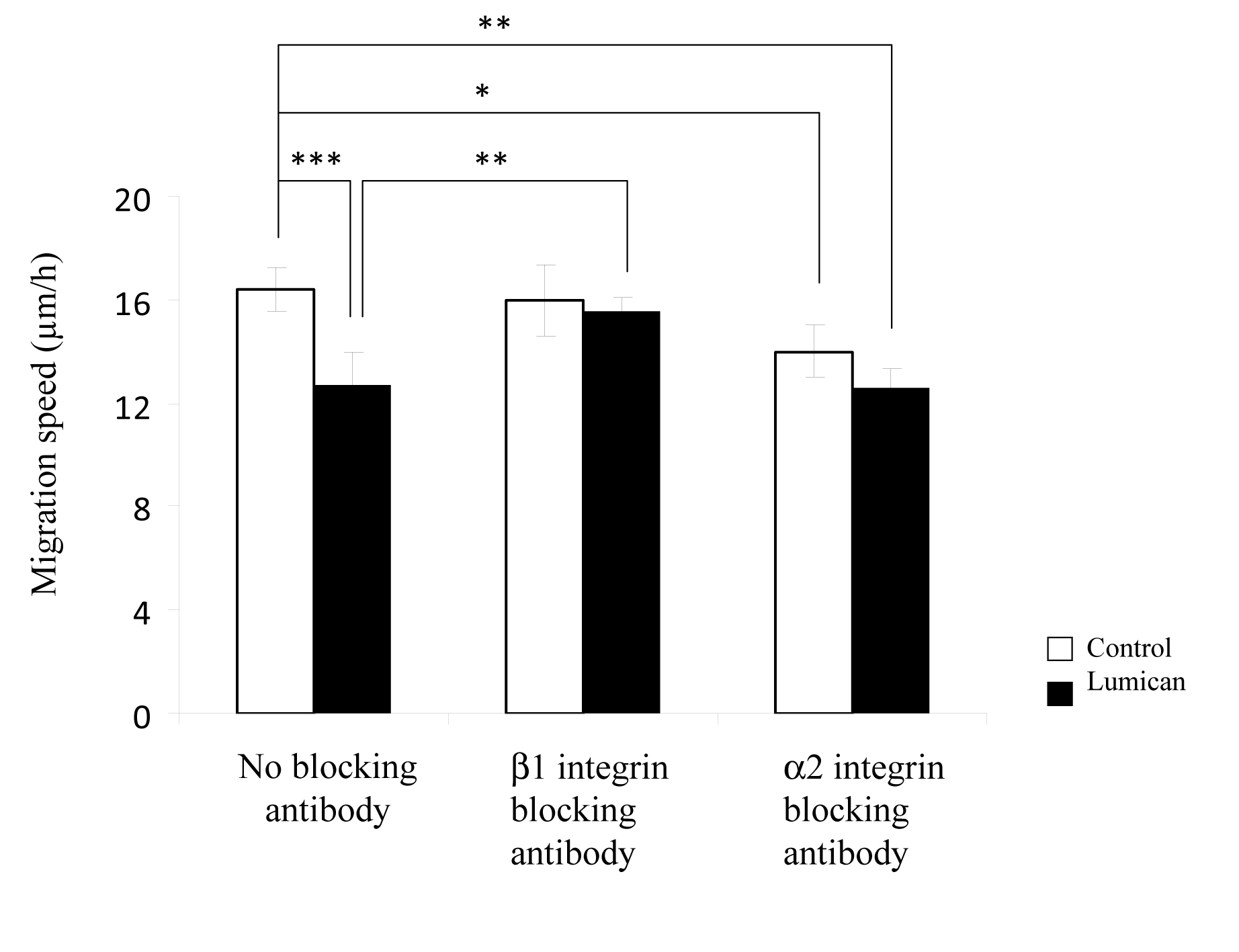

Supplement: Figure S6 — Effect of blocking antibodies raised against human β1 and α2 integrin subunits on MSC migration in presence or absence of lumican. Blocking antibodies (10 µg/ml) anti-human α2 (MAB 1950) and β1 (MAB 1951) integrin subunits were incubated with MSC during 24 h in vitro wound healing assays in presence or absence of 100 nM lumican. The cell migration was video-recorded and the migration speed was measured as described in the materials and methods section. The results are representative of the two independent experiments. Data are expressed as means ± SD. (*p<0.05, **p<0.01, ***p<0.001). (TIF) [file pone.0050709.s006.tif]
