# Supplementary material for: Effect of Lumican on the Migration of Human Mesenchymal Stem Cells and Endothelial Progenitor Cells: Involvement of Matrix Metalloproteinase-14
Source: PLoS One. 2012 Dec 7;7(12):e50709. doi: 10.1371/journal.pone.0050709 (PMC3517548; doi:10.1371/journal.pone.0050709)
Supplement: Material S1 — Supplemental Materials. (DOC) [file pone.0050709.s007.doc]

# SUPPLEMENTAL MATERIALS

Supplemental materials include full methods and legends of supplemental materials.

Figure S1 shows actin cytoskeleton distribution in MSC and EPC seeded on non-coated glass coverslips or coated with type I collagen, fibronectin, and lumican. Figure S2 shows the expression in MSC and EPC of FAK-pY397 and total FAK by Western immunoblotting after 15 min incubation with 100 nM of lumican as compared to control. Figure S3 shows lumican effect on proliferation and apoptosis of MSC and EPC. Figure S4 shows Gallardin® effect on MSC and EPC tube-like formation. Figure S5 shows lumican effect on dermal fibroblast tube-like formation. Figure S6 shows MSC migration during 24h incubation with anti-human β1 and α2 integrin subunits blocking antibodies in presence or absence of 100 nM lumican.

Supplemental Table S1 shows top 12 genes down-regulated and up-regulated in EPC *versus* MSC. Supplemental Table S2 shows a list of primers used for real time PCR reaction.

**Materials and Methods**

Reagents

Recombinant human lumican core protein (37 kDa) and its glycosylated form (57 kDa) were produced as previously described [1,2]. Type I collagen was prepared from rat tail tendon [3]. Human plasma fibronectin and laminin were purchased from Millipore (Molsheim, France). Hoechst 33342 was obtained from Invitrogen (Cergy-Pontoise, France). Matrigel® was purchased from BD Biosciences (Bedford, MA, USA). VEGF was purchased from Promocell (Heidelberg, Germany) and basic Fibroblast Growth Factor (bFGF) from Sigma (St Quentin Fallavier, France). Recombinant TIMP-1, -2, -3 were purchased from R&D Systems (Minneapolis, MN, USA). N-glycosidase F (PNGaseF) was purchased from Roche Diagnostics (Mannheim, Germany).

The following rabbit polyclonal primary antibodies were used: anti-human cyclin D1 (SP4, Labvision, CA, USA), anti-human Bax and anti-human Bcl-2 (Cell Signaling Technology, USA), anti-human MMP-14 (Abcam, Cambridge, UK), and anti-human von Willebrand Factor and anti-human β1 integrin (AB1952P) (Millipore). Anti-human CD31, anti-human total focal adhesion kinase (FAK) and anti-human actin were purchased from Santa Cruz Biotechnology (Heidelberg, Germany).

The following mouse monoclonal antibodies were used: anti-human MMP-14 directed against the catalytic domain (Mab 3329), anti-human β1 integrin (blocking antibody, MAB 1951), anti-human α2 integrin (blocking antibody, MAB 1950) were all purchased from Millipore, mouse isotype control IgG1κ fraction (MOPC-21) from Sigma, anti-human FasR from Santa Cruz Biotechnology, anti-human α2 integrin (MAB 611016) and anti-human FAK-pY397 from BD Biosciences.

The following phycoerythrin (PE) conjugated mouse monoclonal antibodies were used in flow cytometry assays: anti-CD45 and IgG1 isotype control were purchased from Beckman Coulter (Paris, France), anti-human CD73, anti-CD90 and IgG1 k isotype control were purchased from BD Biosciences.

Cell Culture

MSC were prepared in the Unité de Thérapie Cellulaire et banque de Tissus (CHU Brabois, Vandoeuvre Lès Nancy, France). Bone marrow samples, aspirated from the iliac crest, were obtained from healthy donors (aged between 4-45 years old), following informed consent. Bone marrow cells were initially plated in αMEM medium (Lonza, Verviers, Belgium) at the concentration of 7.5×10^4^/cm^2^. After 4-48 hours, growth medium and non-adherent cells were discarded. MSC were expanded in αMEM medium supplemented with 1.2 µg/µl bFGF. At 80% of confluence, cells were trypsinized with a Detach Kit (Promocell). MSC were cultured up to five passages.

EPC were obtained from 7 day-old semi-confluent MSC by incubation in endothelial differentiation medium consisting of Endothelial cell Basal Medium-2 with supplement pack (EBM-2 medium, Promocell) and 50 ng/ml of VEGF. Cells were allowed to differentiate for 13 days under these conditions with a regular change of the VEGF-supplemented EBM-2 medium every 3 days [4-6].

Cells were seeded on uncoated culture plates or on different coatings of ECM proteins: fibronectin (10 µg/ml), laminin (10 µg/ml), recombinant human lumican (30 µg/cm^2^), or type I collagen (30 µg/cm^2^). Trypan blue exclusion assay was used to check the viability of the cells.

Immunophenotyping by Flow Cytometry

Approximately 10^5^ MSC or EPC were incubated with 10 µL of monoclonal primary antibody conjugated with PE: CD45, CD73, CD90, vWF, IgG1 isotype control and IgG1κ isotype control. The samples were incubated for 30 min at 4°C. The samples were then analyzed by flow cytometry (FACScalibur, BD Biosciences) with CellQuest® software.

# Scanning Laser Confocal Microscopy

Immunolabelling was performed as already described [7]. For the detection of actin cytoskeleton, cells were permeabilized with 0.1 % Triton X-100 and incubated 1h at room temperature with Alexa Fluor®488-conjugated phalloidin. Slides were observed under confocal laser scanning microscope (Zeiss LSM 700).

Whole Genome Expression Profiling Experiments

Transcriptome profiles of MSC and EPC were analyzed using Affymetrix GeneChip® Human Gene 1.0 ST microarrays (Santa Clara, CA, USA). Total RNA from three MSC donors and the corresponding *in vitro* differentiated-EPC were extracted using Qiagen RNeasy kit (Hilden, Germany) following manufacturer’s instructions. The RNA quality was assessed using RNA 6000 Nanochips with the Agilent 2100 Bioanalyzer (Agilent, Paolo Alto, USA). Single cDNA was synthesized from 300 ng of total RNA using the GeneChip® Whole Transcript (WT) cDNA synthesis and amplification kit as recommended by the manufacturer (Affymetrix, instruction manual P/N 701880 Rev. 4). The sense cDNA was then fragmented by uracil DNA glycolase (UDG) and apurinic/apyrimidic endonuclease 1 (APE1) and biotin-labelled with terminal deoxynucleotidyl transferase (TdT) using the GeneChip® WT terminal labelling kit (Affymetrix). Hybridization was performed by incubating 2.4 µg of biotinylated target onto the array at 45°C for 17 hours with a permanent rotation of 60 rpm. Arrays were washed and stained using the GeneChip® hybridization, wash and stain kit and the GeneChip® fluidics station 450 (Affymetrix). The arrays were then scanned using the GeneChip® scanner 3000 7G (Affymetrix) and raw data were extracted from the scanned images and quality checked using the Affymetrix expression console software. Microarray data are available in the ArrayExpress database ([www.ebi.ac.uk/arrayexpress](http://www.ebi.ac.uk/arrayexpress/)) under accession number E-MEXP-3071.

Standard pipeline from the Partek® Genomics Suite^TM^ (Partek, St Louis, USA) software was used for further analysis of expression CEL data files. Background correction, quantile-quantile normalization and probe set summarization were performed using the Robust Multichip Analysis (RMA) method [8]. Statistical analysis was performed using the Analysis of Variance (ANOVA) approach in Partek GS. Only genes showing a significant differential expression (False Discovery Rate (FDR) < 0.05) and a fold change > 1.5 were selected for further analysis.

Functional analysis of microarray data was performed using the Ingenuity Pathway analysis (IPA Ingenuity Systems, Inc, Redwood City, CA, USA) software. Genes found differentially expressed with a FDR < 0.05 were submitted to the Ingenuity knowledge proprietary database to identify the biological functions, canonical pathways and networks that were most significant to the dataset. Fisher’s exact test was used to estimate the significance of the incidence of pathways. A *p* value ≤ 0.05 was considered as statistically significant and indicated a non-random enrichment of the experimental dataset by members of a specific pathway as compared to all genes represented on the Affymetrix arrays.

*In vitro* Proliferation Assays

MSC and EPC growth was determined for cells (1×10^4^ cells/well) seeded on uncoated or lumican-coated (30 µg/cm^2^) 6-well plates for 7 days (MSC) or 21 days (EPC). The cell proliferation in the presence of the lumican substratum was then analyzed using 3-[4,5-dimethylthiazol-2-yl]-2,5-diphenyltetrazolium bromide (MTT) (Sigma). For this purpose, cells were incubated with complete culture medium supplemented with 0.5 mg/ml MTT for 3 hours at 37°C. MTT solution was then replaced by DMSO and absorbance at 560 nm was measured.

Hoechst 33342 staining

# Sterile glass coverslips, 12 mm in diameter, were coated with either type I collagen (30 µg/cm^2^), fibronectin (10 µg/ml), or lumican (30 µg/cm^2^). Non-coated coverslips were used as controls. Cells were grown to 80% confluence on coverslips for 24 h. Hoechst 33342 (Invitrogen), at a concentration of 5 µg/mL, was applied for 10 min to stain the nuclei. Slides were observed under confocal laser scanning microscope (Zeiss LSM 700) and photographed.

Western Blotting Analysis

MSC or EPC seeded for 7 days or 21 days, respectively, on the different ECM coatings were washed twice with PBS to remove residual FBS. After 24 h of starvation in a 6-well plate coated with type I collagen (30 µg/cm^2^), fibronectin (10 µg/ml), laminin (10 µg/ml) or lumican (30 µg/cm^2^) cell culture media were collected. Cell monolayers were scrapped and lysed in cell lysis buffer [50 mM Tris-HCl (pH 7.6), 0.5 M NaCl, 0.02% NaN_3_, 0.6% NP40, 5 mM EDTA, 1 mM iodoacetamide, 1 mM PMSF]. The protein concentration was determined by Bradford method [9]. When needed, the protein extracts were incubated for 16 h at 37°C with 2 units of N-glycosidase F.

Total cell proteins (30µg) were subjected to electrophoresis in a 0.1 % SDS, polyacrylamide gel and proteins were transferred onto Immobilon-P membranes (Millipore) by electroblotting. The membranes were soaked in TBS-T solution (0.1% Tween 20, 20 mM Tris and 140 mM NaCl, pH 7.6) containing 5% nonfat milk (BioRad) for 2h at room temperature. After washing, the membranes were incubated with primary antibodies overnight at 4°C at the following dilutions: anti-CD31 (1:1000), anti-α2 integrin (1:500), anti-β1 integrin (1:1000), anti-MMP-14 (1:5000), anti-cyclin D1 (1:500), anti-Bax (1:1000), anti-Bcl-2 (1:1000), anti-Fas receptor (FasR) (1:500), total FAK (1:1000), and FAK-pY397(1:1000), anti-actin (1:500).

The membranes were washed with TBS-T and probed with a 1:10000 dilution of a corresponding secondary antibody conjugated to horseradish peroxidase in a solution of 1% nonfat milk in TBS-T for 30 min at room temperature. After washing in TBS-T, the bands were revealed by the ECL Plus Chemoluminescence Detection kit (GE Healthcare, Orsay, France). Membranes were scanned on a Vilber Lourmat (Marne-la-Vallée, France) imaging and gel documentation system. Bax and Bcl-2 protein expression levels were quantified and the Bax/Bcl-2 ratio was determined.

Gelatin Zymography

To determine MMP-2, MMP-9 activities, MSC or EPC cell lysates or cell-conditioned media were analyzed on SDS-polyacrylamide gels containing 1 mg/mL gelatin. Recombinant MMP-2 and MMP-9 (Millipore) were used as markers. The gels were stained with Coomassie Brillant Blue G-250 (Sigma) and MMP activities were detected as transparent bands on the blue background.

Quantitative Real Time PCR

Total RNA of MSC was isolated using RNeasy® Plus Mini Kit (Qiagen, Courtaboeuf, France) based on the guanidine thiocyanate method according to manufacturer's instructions. Determination of RNA quality was realized on an Agilent 2100 Bioanalyzer (Agilent Technologies, Massy, France) using the RNA 6000 Nano Assay according to the manufacturer’s instructions and using the Agilent 2100 Bioanalyzer Software. Reverse transcription was performed with 1 μg of total RNA in a total volume of 20 μL of a mix containing 200 U of M-MLV reverse transcriptase (Invitrogen), 250 ng random hexamers, 0.5 mM mix of dNTP, 40 U RNaseOut Ribonuclease Inhibitor (Invitrogen), 10 mM dithiothreitol, 10 mM MgCl_2_, 50 mM KCl, 20 mm Tris/HCl, pH 8.4. The reaction was performed at 42°C for 45 min.

Real-time PCR experiments were performed using SYBR® Green I as the intercalating agent. Each 25 μL PCR contained cDNA template, SYBR® *Premix ExTaq*™ (TaKaRa), ROX® Reference Dye II and 0.2 μM of each gene-specific primer. PCR was performed on a Mx3005P thermocycler (Agilent Technologies, Massy, France) using pairs of specific primers for MMP-1, -2, -9, -13, -14, -15, -16 and TIMP-1, -2, -3, -4 genes. Primer sequences and size of the PCR product for each targeted gene are described in supplemental material (Table S2). The specificity of PCR amplification products was assessed by dissociation melting-curve analysis. After the reaction was completed, Ct value was calculated from the amplification plots. The standard curves were generated with serially diluted solutions (1/10 – 1/100000) of cDNA from MSC cells. Each sample was normalized simultaneously to EEF1A1 and RPS29 housekeeping gene transcript content. The ΔΔCt method was used for the relative quantification. PCR assays were conducted in triplicate for each sample on four different donors.

Transfection of MSC

Nucleofection of MSC was performed using Amaxa™ Nucleofector™ Technology [10], according to the optimized protocol provided by the manufacturer (Lonza Verviers, Belgium). Briefly, cells were gently resuspended in 100 µl of Human Mesenchymal Cells Nucleofector Solution (Lonza), mixed with 2 µg plasmid DNA (pGFP only or pGFP containing recombinant human MMP-14 gene), and pulsed with the U-23 program. Immediately after, cells were transferred into pre-warmed fresh medium in six-well plates. The efficiency of transfection was determined 48h (Western blotting) and 72 hours (GFP-positive cell counts) after nucleofection. MSC were transiently transfected with MMP-14-GFP or GFP only (mock) vectors with the efficiency of 40.43 ± 12.58% and 48.31 ± 3.79%, respectively.

*In vitro* Migration Assays

The migration assay was done using culture-inserts (Biovalley, Marne-la-Vallée, France) composed of 2 chambers separated by a “wall”. After withdrawing of the insert, the empty space left by the “wall” simulates a wound and enables the cells to migrate.

To determine the effect of MMP-14 overexpression, mock or MMP-14-GFP-transfected MSC were harvested 48 hours post-transfection and seeded on glass bottom 12-well plates (MatTek Corp.) in culture-inserts (15x10^3^ cells per chamber) in serum-free cell culture medium. Twenty four hours after incubation at 37°C and 5% CO_2_, the culture inserts were removed, cells were rinsed twice with PBS and the wells were filled with 2 mL of serum-free cell culture medium. When needed, lumican (57 kDa) was added at a final concentration of 100 nM.

Motility of GFP-positive MSC was determined by means of computer-assisted phase contrast and fluorescence videomicroscopy (Axiovert 200M; Zeiss, Oberkoken, Germany) equipped with a small transparent environmental chamber (Climabox; Zeiss) with 5% (v/v) CO_2_ in air at 37°C. The microscope was driven by the Metamorph® Software (Roper Scientific, Evry, France), and images were recorded with a charge-coupled device camera (CoolsnapHQ; Roger Scientific) during 24 hours at 1 hour intervals. Cell migration (10 GFP-positive single cells per microscopic field, 4 microscopic fields per insert, 3 replicate inserts for each condition) was characterized and quantified using an interactive tracking method as already described [11].

Blocking antibodies (10 µg/ml) against human α2 integrin (MAB 1950, Millipore) and human β1 integrin (MAB 1951, Millipore) were incubated in MSC culture medium during the *in vitro* wound healing assays. The cell migration was video-recorded for 24h and the migration speed and the trajectories were measured as described above.

*In vitro* Invasion Assays

ThinCert™ cell culture inserts (24-well, pore size 8 μm; Greiner Bio-One, Courtaboeuf, France) were seeded with 50,000 mock or MMP-14-GFP-transfected MSC 48 hours post-transfection in 200 μL of αMEM medium containing 0.5% BSA. For studies involving 57 kDa lumican (100 nM), the protein was applied to the upper chamber at the time of seeding. Inserts were pre-coated with 50 µg of growth factor reduced Matrigel® (BD Biosciences) (gelled at 37ºC for 1h). Eight hundred μL of medium with 10% FBS were added to the lower chamber and served as a chemotactic agent for MSC. Negative control medium contained 2% BSA. After 48h of incubation, non-invading cells were wiped off from the upperside of the membrane and cells on the lower side were fixed in 4% paraformaldehyde (20 min at room temperature). Invasion of MSC was determined by counting the number of Hoechst 33342 (5 µg/mL, Invitrogen)-stained nuclei on the lower side of the membrane under ×200 magnification using a Zeiss Axiovert-25 inverted microscope equipped with a digital camera (Carl Zeiss). Each individual experiment (n=3) had triplicate inserts and three microscopic fields were counted per insert.

**Legends to Supplementary Figures**

**Figure S1. Actin cytoskeleton distribution in MSC and EPC seeded on non-coated glass coverslips or coated with type I collagen, fibronectin, and lumican.**

MSC (a-d) and EPC (e-h) were grown to 80% confluence for 24 h on glass coverslips (a, e) pre-coated with 30 µg/cm^2^ type I collagen (b, f), 10 µg/ml fibronectin (c, g), or 30 µg/cm^2^ lumican (d, h). The distribution of actin cytoskeleton was not significantly altered in MSC and EPC in presence of lumican as compared to other ECM substrata. Scale bar: 20 µm.

**Figure S2. Expression of FAK-pY397 and total FAK in MSC and EPC.**

Cells were grown to confluence on 6-well plate. Expression of FAK-pY397 and total FAK in MSC and EPC was analyzed by Western immunoblotting after 15 min incubation without or with 100 nM of lumican. The levels of FAK-pY397 and total FAK were quantified by densitometric analysis and the FAK-pY397/FAK ratios were determined.

**Figure S3. Effect of lumican on the proliferation and the apoptosis of MSC and EPC.**

(A): Proliferation assay of MSC and EPC cultured on plastic or lumican-coated (30 µg/cm^2^) 6-well plates for 7 or 21 days, respectively. The results were reported as mean values (O.D. 560) ±S.D. (n=3). (B): Cells were grown to 80% confluence for 24 h on coverslips pre-coated with type I collagen (30 µg/cm^2^), fibronectin (10 µg/ml), or lumican (30 µg/cm^2^). The cell cultures were stained with Hoechst 33342. Scale bar: 20 µm. (C): Semi-confluent cells were maintained for 7 days (MSC) or 21 days (EPC), in presence of different ECM coatings: type I collagen (30 µg/cm^2^), fibronectin (10 µg/ml), recombinant human lumican (30 µg/cm^2^). Cyclin D1, Bax, Bcl-2, FasR, and actin protein expression in MSC and EPC was then analyzed by Western blotting as described in Materials and Methods. (D): The levels of Bax and Bcl-2 were quantified by densitometric analysis and Bax/Bcl-2 ratios were determined. These results are representative of three independent experiments. Data are expressed as means ± S.D.

**Figure S4. Gallardin® effect on MSC and EPC tube-like formation.**

Tube formation on Matrigel® (a-d) in control (a, c) or 10^-9^ M Gallardin® supplemented medium (b, d) was observed 24 hours after MSC (a, b) and EPC (c, d) seeding. Representative photographs are presented on the left panel. The semi-quantitative evaluation of the tube network from ten randomly selected fields was performed using ImageJ software and NeuronJ plugin (right diagrams). Experiments were performed in triplicate on three different donors. Results represent the mean ± S.D. Scale bar: 180 µm *: *p*<0.05.

**Figure S5. Lumican effect on dermal fibroblast tube-like formation.**

Tube formation on Matrigel® in control (a) or 100 nM lumican supplemented medium (b) was observed 24 hours after dermal fibroblast seeding (a, b). Representative photographs are presented on the left panel. The semi-quantitative evaluation of the tube network from ten randomly selected fields was performed as described above (right diagrams). Experiments were performed in triplicate on three different donors. Results represent the mean ± S.D. Scale bar: 180 µm.

**Figure S6. Effect of blocking antibodies raised against human β1 and α2 integrin subunits on MSC migration in presence or absence of lumican.**

Blocking antibodies (10 µg/ml) anti-human α2 (MAB 1950) and β1 (MAB 1951) integrin subunits were incubated with MSC during 24h *in vitro* wound healing assays in presence or absence of 100 nM lumican. The cell migration was video-recorded and the migration speed was measured as described in the materials and methods section. The results are representative of the two independent experiments. Data are expressed as means ± SD. (*: *p*<0.05, **: *p*<0.01, ***: *p*<0.001).
