# Supplementary material for: Effect of Lumican on the Migration of Human Mesenchymal Stem Cells and Endothelial Progenitor Cells: Involvement of Matrix Metalloproteinase-14
Source: PLoS One. 2012 Dec 7;7(12):e50709. doi: 10.1371/journal.pone.0050709 (PMC3517548; doi:10.1371/journal.pone.0050709)
Supplement: Table S1 — Top 12 genes down-regulated and up-regulated in EPC versus MSC. (DOC) [file pone.0050709.s008.doc]

Table S1. Top 12 genes down-regulated and up-regulated in EPC *versus* MSC

| **Fold change down-regulated** | | | |  | **Fold change up-regulated** | | | |
| --- | --- | --- | --- | --- | --- | --- | --- | --- |
| Gene | Accession number | Exp. Value | Gene product function |  | Gene | Accession number | Exp. Value | Gene product  function |
| MST131 | AF176921 | -14.775 | growth and development; signal transduction |  | APOD | NM_001647 | +29.830 | lipoprotein metabolism |
| SFRP4 | NM_003014 | -13.347 | signal transduction |  | FKBP5 | NM_001145775 | +12.891 | immunoregulation,  protein folding and trafficking |
| PDE3A | NM_000921 | -10.184 | signal transduction |  | CPM | NM_001874 | +12.469 | monocyte to macrophage differentiation |
| CXCL12 | NM_000609 | -10.121 | lymphocytes chemotaxis |  | C13orf15 | NM_014059 | +7.173 | cell cycle progression |
| KRT7 | NM_005556 | -9.427 | structural protein |  | FAM40B | NM_020704 | +5.697 | cell shape, actin filament distribution, cell migration |
| PTGIS | NM_000961 | -9.237 | vasodilation, hemostasis |  | STEAP1 | NM_012449 | +5.238 | cell-cell communication |
| DACT1 | NM_016651 | -8,742 | signal transduction |  | DIRC1 | NM_052952 | +4.148 | unknown |
| NRXN3 | NM_004796 | -8.170 | cell-cell communication |  | PROS1 | NM_000313 | +3.851 | coagulation/hemostasis, inflammation |
| COMP | NM_000095 | -7.988 | adhesion, structural protein |  | TMEM171 | NM_173490 | +3.615 | unknown |
| BST1 | NM_004334 | -7.275 | pre-B-cell growth |  | PHC2 | NM_198040 | +3.032 | regulatory, transcription factor |
| VCAN | NM_004385 | -5.289 | cell adhesion, proliferation, migration and angiogenesis |  | RPS24 | NM_033022 | +2.775 | translation, protein synthesis |
| NEK7 | NM_133494 | -4.821 | mitosis |  | ZFP36L1 | NM_004926 | +1.999 | transcription |
| **MST131:** 44-kD glycoprotein interacting with the NEU/ERBB2 receptor tyrosine kinase; **SFRP4**: Secreted Frizzeled-related protein 4; **PDE3A**: Phosphodiesterase 3A; **CXCL12:** Stromal cell-derived factor 1**; KRT7**: Keratin 7; **PTGIS**: Prostaglandin I2 synthase; **DACT1**: Dapper; **NRXN3**: Neurexin III; **COMP**: Cartilage oligomeric matrix protein EDM1; **BST1**: Bone marrow stroma cell antigen 1; **VCAN**: Versican; **NEK7**: NIMA-related kinase 7 | | | |  | **APOD**:Apolipoprotein D; **FKBP5**: FK506 binding protein 5; **CPM**: Carboxypeptidase M; **C13orf15**: Chromosome 13 open reading frame 15; **FAM40B:** Family with similarity 40 member B (dermal lymphatic endothelial cells); **STEAP1**: Six transmembrane epithelial antigen of the prostate; **DIRC1**: Disrupted in renal carcinoma 1; **PROS1**: Protein S (alpha) AW214361; **TMEM171**: Transmembrane protein 171; **PHC2**: Polyhomeotic-like2; **RPS24**: Ribosomal protein S24; **ZFP36L1**: Zinc finger protein 3 | | | |
