# Supplementary material for: Effect of Lumican on the Migration of Human Mesenchymal Stem Cells and Endothelial Progenitor Cells: Involvement of Matrix Metalloproteinase-14
Source: PLoS One. 2012 Dec 7;7(12):e50709. doi: 10.1371/journal.pone.0050709 (PMC3517548; doi:10.1371/journal.pone.0050709)
Supplement: Table S2 — List of primers used for quantitative real time PCR reaction. (DOC) [file pone.0050709.s009.doc]

Table S2. List of primers used for quantitative real time PCR reaction

| **Primer** | **Sequence (5'→3')** | **Amplicon size (bp)** | **Accesion number** |
| --- | --- | --- | --- |
| MMP-1 | F- GCTTTCCTCCACTGCTGCT | 144 | [NM_002421.3](http://www.ncbi.nlm.nih.gov/nucleotide/225543092?report=genbank&log$=nucltop&blast_rank=3&RID=13RD8PS701N) |
|  | R- CTTGCCTCCCATCATTCTTC |  |  |
| MMP-2 | F- TCTTCCCCTTCACTTTCCTG | 111 | [NM_004530.4](http://www.ncbi.nlm.nih.gov/nucleotide/189217851?report=genbank&log$=nucltop&blast_rank=2&RID=13RY1M0B01N) |
|  | R- ACTTGCGGTCGTCATCGT |  |  |
| MMP-9 | F- TGACAGCGACAAGAAGTGG | 203 | [NM_004994.2](http://www.ncbi.nlm.nih.gov/nucleotide/74272286?report=genbank&log$=nucltop&blast_rank=1&RID=13S7K1T301S) |
|  | R- GGCGAGGACCATAGAGGTG |  |  |
| MMP-13 | F- CAGTCTTTCTTCGGCTTAGAGG | 240 | [NM_002427.3](http://www.ncbi.nlm.nih.gov/nucleotide/296010793?report=genbank&log$=nucltop&blast_rank=1&RID=13SEPNH501N) |
|  | R- CAGAGGAGTTACATCGGACCA |  |  |
| MMP-14 | F- CGGGTGAGGAATAACCAAGT | 237 | [NM_004995.2](http://www.ncbi.nlm.nih.gov/nucleotide/13027797?report=genbank&log$=nucltop&blast_rank=1&RID=13SM6AV101S) |
|  | R- CCAGAAGAGAGCAGCATCAA |  |  |
| MMP-15 | F- CCAAGAGGACAGGTACTGGCGCTT | 189 | [NM_002428.2](http://www.ncbi.nlm.nih.gov/nucleotide/45594662?report=genbank&log$=nucltop&blast_rank=1&RID=13ST4X1K01N) |
|  | R- ATCCGCAGGCGCTCATTGTCG |  |  |
| MMP-16 | F- CAATTGACTGGATGAAGAAGCCCCG | 204 | [NM_005941.4](http://www.ncbi.nlm.nih.gov/nucleotide/149999366?report=genbank&log$=nucltop&blast_rank=2&RID=13T0J0C701S) |
|  | R- GCCACACATCAAAGGCACGGC |  |  |
| TIMP-1 | F- GACGGCCTTCTGCAATTCC | 78 | [NM_003254.2](http://www.ncbi.nlm.nih.gov/nucleotide/73858576?report=genbank&log$=nucltop&blast_rank=1&RID=15TVHPPS014) |
|  | R- GTATAAGGTGGTCTGGTTGACTTCTG |  |  |
| TIMP-2 | F- GTGACCCAGTCCATCCAGAG | 128 | [NM_003255.4](http://www.ncbi.nlm.nih.gov/nucleotide/73858577?report=genbank&log$=nucltop&blast_rank=1&RID=15U91K1A014) |
|  | R- GAGCACCACCCAGAAGAAGA |  |  |
| TIMP-3 | F- GCAGATAGACTCAAGGTGTGTGAAA | 102 | [NM_000362.4](http://www.ncbi.nlm.nih.gov/nucleotide/75905820?report=genbank&log$=nucltop&blast_rank=1&RID=15TYMRFD014) |
|  | R- TCCCTCACTCTTACATGCAGACA |  |  |
| TIMP-4 | F- AGGACCTGTCCTTGGTGCAGA | 196 | [NM_003256.2](http://www.ncbi.nlm.nih.gov/nucleotide/48255910?report=genbank&log$=nucltop&blast_rank=1&RID=15U3NBBX016) |
|  | R- GCCGTCAACATGCTTCATACAGA |  |  |
| RPS29 | F- aagatgggtcaccagcagctctactg | 70 | NM001030001 |
|  | R- AGACGCGGCAAGAGCGAGAA |  |  |
| EEF1A1 | F- CTGGAGCCAAGTGCTAATATGCC | 222 | NM001402 |
|  | R- CCAGGCTTGAGAACACCAGTC |  |  |

EEF1A1 - eukaryotic translation elongation factor 1 alpha 1 ; MMP – matrix metalloproteinase; RPS29 - 40S ribosomal protein S29; TIMP – tissue inhibitor of metalloproteinase
